# Uncovering the bequeathing potential of apoptotic mesenchymal stem cells via small extracellular vesicles for its enhanced immunomodulatory and regenerative ability

**DOI:** 10.1186/s13287-025-04370-x

**Published:** 2025-06-07

**Authors:** Meenakshi Mendiratta, Mohini Mendiratta, Yashvi Sharma, Ranjit Kumar Sahoo, Neena Malhotra, Sujata Mohanty

**Affiliations:** 1 Stem Cell Facility, DBT-Centre of Excellence for Stem Cell Research, All India Institute of Medical Sciences, New Delhi, 110029 India; 2 Department of Medical Oncology, Dr. B. R. Ambedkar Institute Rotary Cancer Hospital, All India Institute of Medical Sciences, New Delhi, 110029 India; 3 Department of Obstetrics & Gynaecology, All India Institute of Medical Sciences, New Delhi, 110029 India

**Keywords:** Mesenchymal stem cells, Apoptotic small extracellular vesicles, Efferocytosis, Immunomodulatory, Antioxidant, Bone marrow, Wharton’s Jelly

## Abstract

**Background:**

Mesenchymal Stem Cells-derived Small Extracellular Vesicles endowed with regenerative cargo from their parent cells, have emerged as a promising avenue for cell-free therapeutics in regenerative medicine. Notably, deliberate induction of apoptosis in MSCs before sEV isolation has been identified as a strategy to augment the regenerative capabilities of MSCs-sEVs. This study explores a novel approach to enhance the immunomodulatory potential of MSC-sEVs through apoptosis induction and optimal tissue source to ensure consistent and improved clinical outcomes.

**Methods:**

Apoptosis was induced in tissue-specific MSCs using Staurosporine. sEVs^V^ and sEVs^Apo^ were isolated via ultracentrifugation. *Invitro* immune response was assessed via T-cell proliferation, T-regulatory cell induction & macrophage polarization assay. Mitochondrial bioenergetics was studied using MitoSOX staining and Seahorse assay in H2O2-treated HuH7 cells. These findings were validated *invivo* in the CCL4-induced Chronic Liver Disease model via Histopathological staining, biochemical parameters, and fibrotic, pro-inflammatory, and anti-inflammatory markers and assessed the mechanism by targeting TGF-β/SMAD pathway.

**Results:**

Our results demonstrate that sEVs^Apo^ exhibited significantly higher concentrations and superior immunomodulatory effects by suppressing CD3 + T-cell proliferation, promoting T-regulatory cell differentiation, and polarized macrophages towards M2-phenotype. In terms of tissue specificity, it was observed that WJ-sEVs were faring better. sEVs^Apo^ effectively reduced mitochondrial ROS & significantly improved oxidative phosphorylation. *Invitro* findings were corroborated in an *invivo* CLD model, wherein sEVs^Apo^ ameliorated fibrosis and inflammation, by inhibiting TGF-β/ SMAD2/3 pathway.

**Conclusion:**

This study concludes that apoptosis induction can be considered as minimum manipulation strategy to enhance the immunoregulatory and regenerative potential of MSCs-sEVs, thereby expanding their implication in immune disorder.

**Supplementary Information:**

The online version contains supplementary material available at 10.1186/s13287-025-04370-x.

## Background

Mesenchymal stem cells (MSCs) are multipotent stem cells, that can be isolated from various adult tissues, including bone marrow, umbilical cord, adipose tissue, and dental pulp [1, 2]. The attainment of effective clinical outcomes relies on the utilization of MSCs from an optimal tissue source, which can consistently yield improved clinical results, along with posing the least ethical concerns, being easily available & having cost efficacy [3]. MSCs have garnered significant attention in clinical trials due to their regenerative, anti-oxidant, and immunomodulatory properties [4, 5]. These properties are attributed to several mechanisms of action exhibited by MSCs, predominantly including paracrine secretions & efferocytosis by the act of apoptosis [6, 7].

A key modality that has come to light in the context of paracrine secretions by MSCs is the sEVs secreted by them. These vesicles are less than 200 nm in size & are majorly involved in intercellular communication. They carry regenerative cargo including miRNAs, mRNAs, and proteins, but are devoid of nuclei & therefore safer for transplantation as compared to their parent cells [8]. Additionally, sEVs are versatile for diverse formulations and provide convenient storage and transportability [9, 10].

Certain reports have also suggested that transplanted MSCs have a limited lifespan in recipients. They undergo apoptosis either within the host circulation or in the engrafted tissue as a means of getting efferocytosis thereby disseminating their regenerative components [11]. Therefore, this regeneration mechanism has been explored in a few studies by deliberately inducing apoptosis in the MSCs & then validating their regenerative potential [11]. These Apoptotic MSCs (MSCs^Apo^) have been found to possess the ability to induce immunosuppressive effects in animal models of lung injury, liver injury, and spinal cord injury [12, 13]. Pang et al. (2021) in their study, inhibited the apoptotic induction in MSCs by silencing the apoptotic effectors BAK & BAX. They observed that there was a reduction in their immunomodulatory capabilities in a viable form, when administered in an asthmatic mice model, thereby potentiating the need for MSCs for undergoing apoptosis to exert their functional effect [11, 14].

However, employing whole cells for infusion comes with limitations, including the risk of adverse reactions due to the heterogeneity of apoptotic cells, which may also encompass necrotic cells [15, 16]. Consequently, a cell-free approach utilizing Apoptotic Extracellular Vesicles (sEVs^Apo^), including both small & large EVs, has been explored for its potential to mimic therapeutic effects similar to their parent cells [17]. The potential of sEVs^Apo^ has been explored in multiple studies [18–20]. Zheng et al., (2021) observed in their study that Apoptotic Vesicles released by MSCs were able to confer functional modulations to liver macrophages via efferocytosis, and mitigate type 2 diabetes mellitus [21]. This presents a novel insight that sEVs^Apo^ could be a functionally potent modality for targeting immune implicated liver diseases.

While the majority of studies with Viable MSCs (MSCs^V^) have confirmed the superior regenerative potential of small EVs, as compared to the larger EVs, existing studies with MSCs^Apo^ have considered sEVs^Apo^ as a collective heterogenous population of small EVs, large EVs & Apoptotic bodies, which may confer pro- or anti- therapeutic effects due to the packaging of detrimental components synthesized as a part of the cell death process [18–20]. To the best of our knowledge, the comparative functional capabilities of MSCs-sEVs^V^ and MSCs-sEVs^Apo^ are yet to be elucidated. Furthermore, there remains a gap in understanding the optimal source of MSCs for the generation of sEVs^Apo^ in the context of translational applications [19, 20].

Hence, in this investigation, we have thoroughly examined Apoptotic sEVs (sEVs^Apo^) in comparison to Viable sEVs (sEVs^V^) obtained from both Bone Marrow (BM) and Wharton’s Jelly (WJ)-derived MSCs. The objective was to unveil their immunomodulatory, antioxidant, and anti-fibrotic capabilities in an in vitro and in vivo Chronic liver disease model while evaluating apoptosis as a priming strategy to amplify the functional potential of MSCs-sEVs.

## Materials and methods

### Reagents and antibodies

High Glucose (HG)-Dulbecco’s modified Eagle medium (DMEM) and Low Glucose (LG)- DMEM were purchased from Gibco, USA. Fetal Bovine Serum (FBS) was purchased from Himedia, India. STEMPRO^®^ MSC Serum Free Medium CTS, 100X Antibiotic-Antimycotic solution, dead cell apoptosis kit with Annexin V, FxCycle™ PI/RNase Staining Solution kit, Pierce™ BCA Protein Assay Kit were purchased from Thermo Fischer Scientific, USA. Staurosporine, PKH26 Red Fluorescent cell linker kit, Cell Counting Kit-8 kit, pH Rodo Red succinidyl ester, phorbol 12-myristate 13-acetate (PMA), and radioimmunoprecipitation assay lysis buffer was purchased from Sigma, USA. CD-63, ALIX, Flotillin, calnexin, and GAPDH were purchased from Genetex, USA and cleaved caspase-3 was purchased from CST, USA). CD206, Arginase, and iNOS were purchased from eBiosciences, USA. Cell TraceTM CFSE cell proliferation assay kit was purchased from BD Biosciences, US. MitoSOX red was purchased from Life Technologies, USA. Qiazol, PCR starter kit, and miRNeasy mini kit were purchased from Qiagen, Netherlands. SYBR Green Master Mix and Protease inhibitors were purchased from Promega, US. CD105, CD90, CD73, CD29, HLA-class I, HLA-class II, and CD34/45 (BD Pharmingen, France). Phalloidin and TRIzol reagent was purchased from Invitrogen, United States. High-Capacity cDNA Reverse Transcription Kit was purchased from Applied Biosystems, Invitrogen, United States.

### Cell lines

The Human HuH7 and THP 1 cell lines were kindly gifted by the Institute of Liver and Biliary Sciences (ILBS) and the All India Institute of Medical Sciences (AIIMS), New Delhi, India respectively. HuH7 cells were maintained in HG-DMEM supplemented with 10% FBS and 1% Antibiotic-Antimycotic solution. THP 1 and hPBMNCs cells were maintained in RPMI-1640 medium supplemented with 10% FBS and 1% Antibiotic-Antimycotic solution.

### Isolation and characterization of human MSCs

MSCs used in this study were isolated from healthy donors with their written consent after obtaining ethical clearance from the Institutional Committee for Stem Cell Research (IC-SCR) (Ref No. IC-SCR/140/23 (o)), AIIMS, New Delhi. MSCs obtained from the bone marrow (BM-MSCs), and Wharton’s Jelly (WJ-MSCs) were used in this study. The bone marrow was collected from the patient’s donor (*n* = 3) who was undergoing the routine medical test procedure at Dr. B. R. Ambedkar IRCH, AIIMS, New Delhi. Briefly, BM-MSCs were isolated and cultured in LG DMEM containing 10% MSC grade FBS, and 1% Antibiotic-Antimycotic solution at 37 °C in a 5% CO_2_ incubator. Umbilical cord (*n* = 3) was collected in transport media and processed within 24 h of normal or cesarean delivery of the donors with the age group 20–35 years from the Department of Obstetrics and Gynecology, AIIMS, New Delhi. Briefly, the umbilical cord was collected and washed with 1X PBS containing 1% Antibiotics. The cord was cut to expose Wharton’s Jelly part, chopped into small pieces using a surgical blade. The Wharton’s Jelly was placed in 35 mm culture dish containing LG-DMEM with 10% MSC grade FBS and 1% Antibiotic-Antimycotic solution and incubated overnight at 37 °C and 5% CO_2._ MSCs were sub-cultured at 70% confluency and characterized using Flow Cytometry and Trilineage Differentiation. Surface markers CD105, CD90, CD73, CD29, HLA-class I, HLA-class II, and CD34/45 were studied using Flow Cytometry (Beckman Coulter, USA) and analyzed using Kaluza Software Version 2.1. Tri-lineage differentiation (osteocyte, adipocyte, and chondrocyte) was performed as per previously established protocol [1, 22].

To achieve homogeneity, we pooled BM-MSCs (*n* = 3) and WJ-MSCs (*n* = 3) at passage 3 for subsequent experiments.

### Induction of apoptosis in MSCs

Both BM-MSCs and WJ-MSCs were treated with 0.5µM staurosporine (STS) in LG-DMEM for 6, 12 and 24 h [19, 20] The respective cell suspensions were collected, washed, and stained with Annexin V and PI to evaluate the apoptosis rate of apoptotic cells by flow cytometry and analyzed using Kaluza Software Version 2.1 and confirmed by cleaved caspase 3 expression via western blotting.

### Isolation and characterization of apoptotic MSC-derived small extracellular vesicles (sEVs^Apo^)

sEVs^Apo^ were isolated from Apoptotic BM-MSCs (BM-MSCs^Apo^) and Apoptotic WJ-MSCs (WJ-MSCs^Apo^) using a one-step sucrose ultracentrifugation-based method by Optima XPN100 ultracentrifuge in a swinging bucket rotor (Beckman Coulter, USA) [22, 23]. Briefly, MSCs^Apo^ were cultured in STEMPRO^®^ MSC Serum Free Medium CTS for 48 h, and the conditioned medium was collected for the isolation of sEVs^Apo^. The conditioned medium was centrifuged at 300×g for 10 min to remove cellular debris followed by 10,000×g for 30 min to remove microvesicles. The supernatant was collected without disturbing the pellet and added over 30% sucrose solution layer. Then, it was centrifuged at 100,000×g at 4 °C for 90 min. The sucrose layer was collected and washed with phosphate-buffered saline (PBS) at 100,000 × g for 90 min to obtain sEVs^Apo^ pellets. sEVs^Apo^ were resuspended in 500 µL PBS and stored at − 80 °C until further use. sEVs^V^ were isolated from BM-MSCs and WJ-MSCs as per the above-described protocol. BM-sEVs^V^ and WJ-sEVs^V^ were used as a control. sEVs^Apo^ and sEVs^V^ were quantified using a Bicinchoninic Acid (BCA) assay (Pierce™ BCA Protein Assay Kit).

### Characterization of sEVs

The sEVs^V^ and sEVs^Apo^ were characterized as per the MISEV Guideline with the following techniques:

#### Nanoparticle tracking analysis

The sEVs were diluted (1:10) in PBS and acquired for particle concentration and size distribution using NanoSight LM20 (Malvern Instruments Company, United Kingdom). The Brownian motion of each particle was tracked between frames and the size was calculated using the Stokes-Einstein equation [22].

#### Zeta potential

The sEVs were acquired for charge distribution using Malvern Nano zeta sizer (Malvern Instruments Company, United Kingdom). The zeta potential of each particle was tracked between frames.

#### Transmission electron microscopy (TEM)

The sEVs were diluted at a concentration of 1:10 in PBS and placed on Formvar-carbon-coated copper grids and adsorbed for 5 min. The grids were stained with 2% Phosphotungstic acid solution for 1 min. Then, grids were air-dried and observed under the transmission electron microscope (Tecnai, FEI, USA) [22].

#### Western blotting

The sEVs were lysed using radioimmunoprecipitation assay lysis buffer with protease inhibitors. The concentration of sEV lysates was estimated using a BCA protein assay kit. 25 µg of sEVs lysate was subjected to 12% SDS-PAGE gel in reducing conditions for ALIX, calnexin, Cleaved Caspase3, and non-reducing conditions for CD-63. This was followed by transfer to a PVDF membrane (MDI) using a wet transfer system (Bio-Rad, USA). The blot was blocked with 5% BSA in 1×TBS-T solution at room temperature for 1 h followed by incubation with respective primary antibody CD-63 (1:500 dilution), ALIX (1:1000 dilution), calnexin (1:3000 dilution), cleaved caspase 3 (1:1000 dilution) and GAPDH (1:3000 dilution) overnight at 4 °C. The blot was then washed with 1X TBST three times each for five minutes and incubated with HRP-conjugated secondary antibody for 1 h at room temperature. The blot was washed three times using 1XTBS-T and developed using an ECL imager (Bio-Rad, USA) [22].

For liver tissue, total protein was extracted by homogenization in RIPA lysis buffer supplemented with protease inhibitors cocktail. Protein concentrations were quantified using a BCA assay, and 25 µg protein was separated on a 12% SDS-polyacrylamide gel followed by transfer to PVDF membranes. Membranes were blocked with 5% BSA in 1X TBS-T and incubated overnight at 4 °C with primary antibodies [mNTGF-β1, mp-Smad2/3, and mSmad7 (1:1000)]. After washing, membranes were incubated with HRP-conjugated secondary antibodies for 1 h at room temperature and developed using an ECL imager. The relative protein expression levels were normalized to mGAPDH as a loading control, and densitometric analysis was performed using ImageJ software to quantify band intensities.

### Macrophage polarization

To evaluate the immunomodulatory potential of sEVs^Apo^, THP-1 cells were seeded at a density of 1 × 10^6^ cells/well in a 6-well plate. THP-1 cells were differentiated into M0-like macrophages using 25 ng/ml PMA for 24 h in RPMI-1640 medium. M0-like macrophages were then polarized into M1-macrophages by treatment with 10 ng/ml of Lipopolysaccharide (LPS) for 24 h [24]. M1-macrophages were treated with 50 µg/mL BM-sEVs^V^, WJ-sEVs^V^, BM-sEVs^Apo^, and WJ-sEVs^Apo^ for 48 h. The treated macrophages were subsequently characterized with CD206- PE, Arginase 1-APC, and iNOS-FITC antibodies using flow cytometry and analyzed using Kaluza Software Version 2.1.

### In vitro efferocytosis assay

In order to study the uptake of sEVs^Apo^ by the macrophages, we incubated the M1-macrophages with 20ng/ml pH Rodo Red succinidyl ester labeled BM-sEVs^V^, WJ-sEVs^V^, BM-sEVs^Apo^, and WJ-sEVs^Apo^ [11]. Concurrently, M1-macrophages were labeled with 1µM CFSE dye for 20 min. The CFSE stained M1-macrophages were co-cultured with labeled BM-sEVs^V^, WJ-sEVs^V^, BM-sEVs^Apo^, and WJ-sEVs^Apo^ for 48 h. The percentage of sEVs^Apo^ engulfed by M1 macrophages was measured using flow cytometry and analyzed using Kaluza Software Version 2.1.

### T-cell proliferation and differentiation assay

hPBMNCs cells were labeled with 1µM CFSE dye for 20 min followed by stimulation with PHA (5 µg/ml) and IL-2 (50IU/ml) for 48 h. Afterward, stimulated hPBMNCs cells were treated with 50 µg/mL BM-sEVs^V^, WJ-sEVs^V^, BM-sEVs^Apo^, and WJ-sEVs^Apo^ for 3 days. Furthermore, the proliferation and activation capacity of hPBMNCs cells were examined by Cell TraceTM CFSE cell proliferation assay kit and fluorochrome-conjugated antibodies such as CD3, CD4 respectively using a flow cytometer (Beckmann Coulter, US). After 5 days of culture, the percentage of regulatory T cells (CD3^+^CD4^+^CD25^+^FOXP3^+^) was determined by flow cytometer (Beckmann Coulter, US) and analyzed using Kaluza Software Version 2.1 [18].

### Measurement of intracellular reactive oxygen species (ROS)

Intracellular ROS level was measured in cells using MitoSOX red according to the manufacturer’s instructions. Briefly, HuH7 cells were seeded in a 6-well plate at a confluence of 1 × 10^6^ cells/well. Cells were exposed to 100 µM H_2_O_2_ for 30 min. Then cells were washed twice with 1X PBS and treated with 50 µg/mL BM-sEVs^V^, WJ-sEVs^V^, BM-sEVs^Apo^, and WJ-sEVs^Apo^ for 48 h. After treatment, cells were incubated with MitoSOX red at a concentration of 4 µM for 20 min in respective media at 37 °C with 5% CO2 in the dark. The quantification of ROS in terms of the percentage of positive cells was done using a flow cytometer and analyzed using Kaluza Software Version 2.1.

### Cellular bioenergetics studies

Oxygen consumption rate (OCR) was measured using Seahorse XFe24 Extracellular Flux Analyzer (Agilent Technologies, California, USA) serving as an indicator for OXPHOS. HuH7 cells were seeded at a consistent density of 1 × 10^5^/well on CellTak -coated plates (Corning) in XF-Base Media (Agilent Technologies) containing 2.5 mM glucose, 1 mM sodium pyruvate, and 2 mM glutamine and co-cultured with BM- sEVs^V^, WJ- sEVs^V^, BM-sEVs^Apo^, and WJ- sEVs^Apo^ for 48 h. OCR was measured sequentially at the basal level followed by the addition of 1.0 mM oligomycin, 0.75 mM FCCP (fluorocarbonyl cyanide phenylhydrazone), and 0.5 mM rotenone + antimycin to evaluate the changes in mitochondrial respiratory parameters. The plate underwent three measurements at both baseline and post-drugs addition [25]. Data were analyzed using Wave 2.6.1 and GraphPad Prism.Top of Form.

### Quantitative real-time polymerase chain reaction (qRT-PCR)

#### MiRNA expression

Total RNA was extracted from the BM-sEVs^V^, WJ-sEVs^V^, BM-sEVs^Apo^, and WJ-sEVs^Apo^ using Qiazol Reagent and miRNA was isolated using miRNeasy mini kit as per manufacturer’s protocol. The miRNA concentration and purity were measured by a Nanodrop 2000 Spectrophotometer (Thermo Scientific, USA). cDNA was prepared using a PCR starter kit. qRT-PCR was performed in triplicate with a PCR starter kit in CFX96 Real-Time System (Bio-Rad, USA). hsa-miR-16-5p was used as an internal control to normalize the gene expression. hsa-miR-125b-5p, hsa-miR-145b-5p, and hsa-miR-34a expression levels were determined using the 2-ΔΔCt method.

### RNA expression

For the isolation of total RNA from THP1 cells and liver tissue after treatment with BM-sEVs^V^, WJ-sEVs^V^, BM-sEVs^Apo^, WJ-sEVs^Apo^, the total RNA was isolated using the phenol-chloroform extraction method using TRIzol reagent according to the manufacturer’s protocol. Then, 2 µg total RNA was reverse transcribed to give cDNA using the High-Capacity cDNA Reverse Transcription Kit. Quantitative real-time polymerase chain reaction (qRT-PCR) was performed using a CFX96 Real-Time System (Bio-Rad). Glyceraldehyde-3 phosphate dehydrogenase (GAPDH) was used as the housekeeping gene to normalize the gene expression. qRT-PCR was performed in triplicate using SYBR Green Master Mix according to the manufacturer’s instructions. The comparative 2 − ΔΔCt method was performed to evaluate the mRNA expression of IL-1β, TNF-α, IL-10, mCOL-1, mαSMA, mTNF-α, mTGF-β, mIL-10, mIL-6 [22, 26].

### Animals

All experiment protocols were approved by the Institute Animal Ethical Committee (IAEC), AIIMS, New Delhi, India (IAEC Number: 422/IAEC-1/2023). Female C57BL/6 mice, initially six weeks old, were acclimatized for two weeks before the commencement of experiments.

### Establishment of chronic liver disease mice model using carbon tetrachloride and WJ-sEVs^V^ & WJ-sEVs^Apo^ administration

A total of 50 mice were randomly allocated into five groups for analysis on Day 1, Day 5, and Day 12 (*n* = 5 per time point): Group 1 (Healthy/ Sham), Group 2 (CCl4, Untreated), Group 3 (CCl4 + WJ-sEVs^V^, Treated), and Group 4 (CCl4 + WJ-sEVs^Apo^, Treated). To establish a chronic liver disease model, CCl4 (diluted in olive oil, vol/vol) was administered via intraperitoneal injection biweekly for 6 weeks. The dosing schedule was as follows: 0.1 ml/kg for the initial two weeks, 0.2 ml/kg for the subsequent two weeks, and 0.5 ml/kg for the final two weeks to induce chronic liver fibrosis. Sham mice received an equivalent volume of olive oil for 6 weeks [26]. Anaesthesia was not given to mice in the study. After 24 h from the final CCL4 dose, mice were treated with either WJ-sEVs^V^ or WJ-sEVs^Apo^. sEVs via intravenous (i.v.) injection at a concentration of 250 µg in 100 µl of 1X PBS per mouse. The control group was administered 100 µl of 1 X PBS. Mice were euthanized by exposing them to carbon dioxide (CO_2_) in a controlled chamber, and sacrificed on Day 1, Day 5, and Day 12 post-treatment. Liver and blood were collected from each mouse for subsequent analysis.

### Histopathological analysis

Liver tissues were fixed in 10% buffered formalin for 24 h at 4 °C and subsequently processed for paraffin embedding. The 4 μm thick paraffin sections were prepared for further studies. These sections were stained with hematoxylin and eosin (H&E), Sirius Red (SR) staining as well as Masson’s trichrome (MT) staining according to established protocols. To assess the degree of liver fibrosis, digital images of the lesioned area were captured from the Masson trichrome-stained sections for each animal. The area stained for collagen was quantified using ImageJ 1.34 software [26].

### Biochemical analysis

The blood was collected from the retro-orbital sinus of CLD mice. The plasma levels of alanine aminotransferase (ALT), aspartate aminotransferase (AST), and albumin (ALB) were assessed using the Automated Biochemical Analyzer (AU-680, Beckman, Germany) [26].

### Statistical analysis

In this study, all statistical analyses were conducted through GraphPad Prism 8.4.3 software. An unpaired student’s *t-*test was used to compare the two groups. One-way and Turkey’s post hoc tests compared three or more groups. A p-value of < 0.05 was considered statistically significant.

*The work has been reported in line with the ARRIVE guidelines 2.0.

## Results

### MSCs retain their characteristics upon apoptotic induction

MSCs isolated from Bone marrow and Wharton’s jelly exhibited all characteristic features as per International Society of Cell and Gene Therapy (ISCT) guidelines including plastic adherence, fibroblast-like spindle morphology, trilineage differentiation, presence of surface markers CD29, CD90, CD73, CD105, HLA I, and the absence of CD34, CD45, and HLA II (Figure S1).

**Fig. 1:**
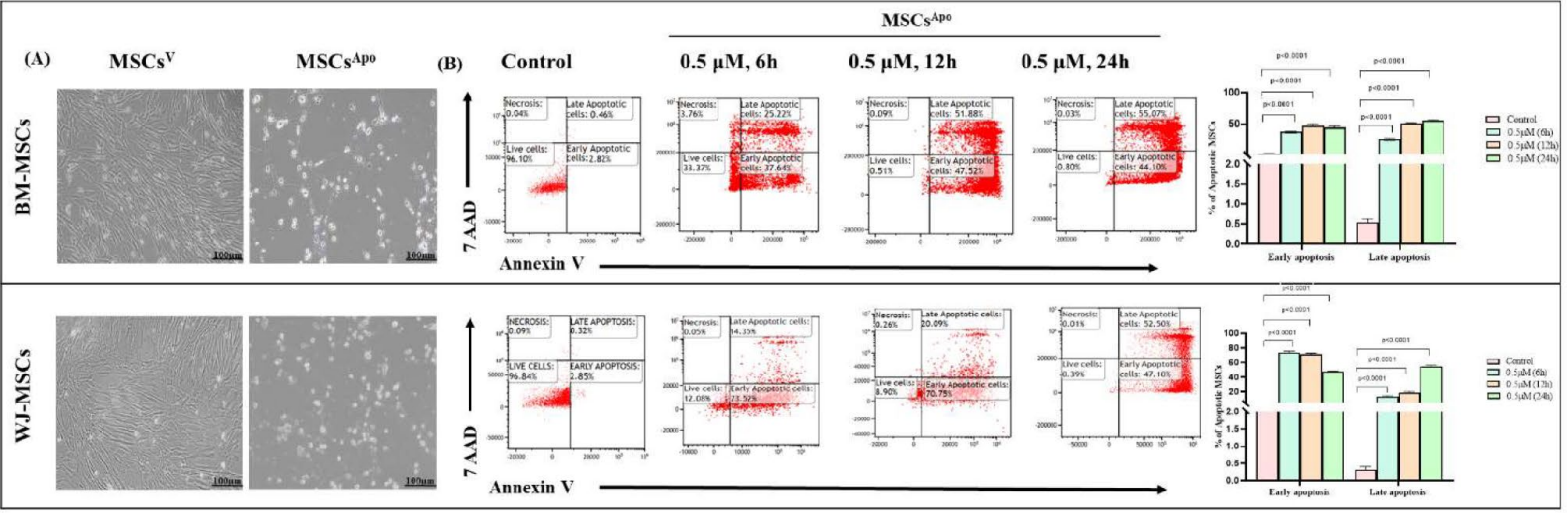
Generation of apoptotic MSCs using Staurosporine (**A**) Representative morphology of Apoptotic Bone Marrow and Wharton’s Jelly MSCs (Scale bar: 100 μm). (**B**) Dot plot representing the percentage of viable, early apoptosis, late apoptosis, and necrotic cells with and without treatment of STS at a dose of 0.5 µM for 6, 12, 24 h. (**C**) The bar graph depicts the percentage of apoptotic MSCs with and without Staurosporine treatment. Data are shown as mean ± SD; Statistical analysis: ****p < 0.0001. All experiments were done in triplicates. Abbreviation- V: Viable; Apo: Apoptotic

MSCs from both sources were subjected to apoptotic induction using 0.5 µM STS for 6 h, 12 h, and 24 h. Annexin/PI staining was used to assess the percentage of cumulative apoptotic cells which was 99.17% (44.10% early & 55.07% late apoptotic cells) and 99.60% (52.50% early & 47.10% late apoptotic cells) at a 24 h time point in BM-MSCs & WJ-MSCs respectively, (Fig. 1B). Furthermore, at this time point, the cells displayed the hallmark features of apoptosis induction including significant morphological alteration, cell blebbing, and shrinkage [27] (Fig. 1A). Induction of apoptosis was also validated via western blotting in both the tissue-specific MSCs in terms of the Cleaved Caspase 3 expression (Figure S2).

Moreover, the immunophenotyping assay with both BM-MSCs^Apo^ & WJ-MSCs^Apo^ validated the maintenance of MSCs characteristics after apoptosis induction (Figure S2).

### sEVs^Apo^ maintain similar characteristics to sEVs^V^

**Fig. 2:**
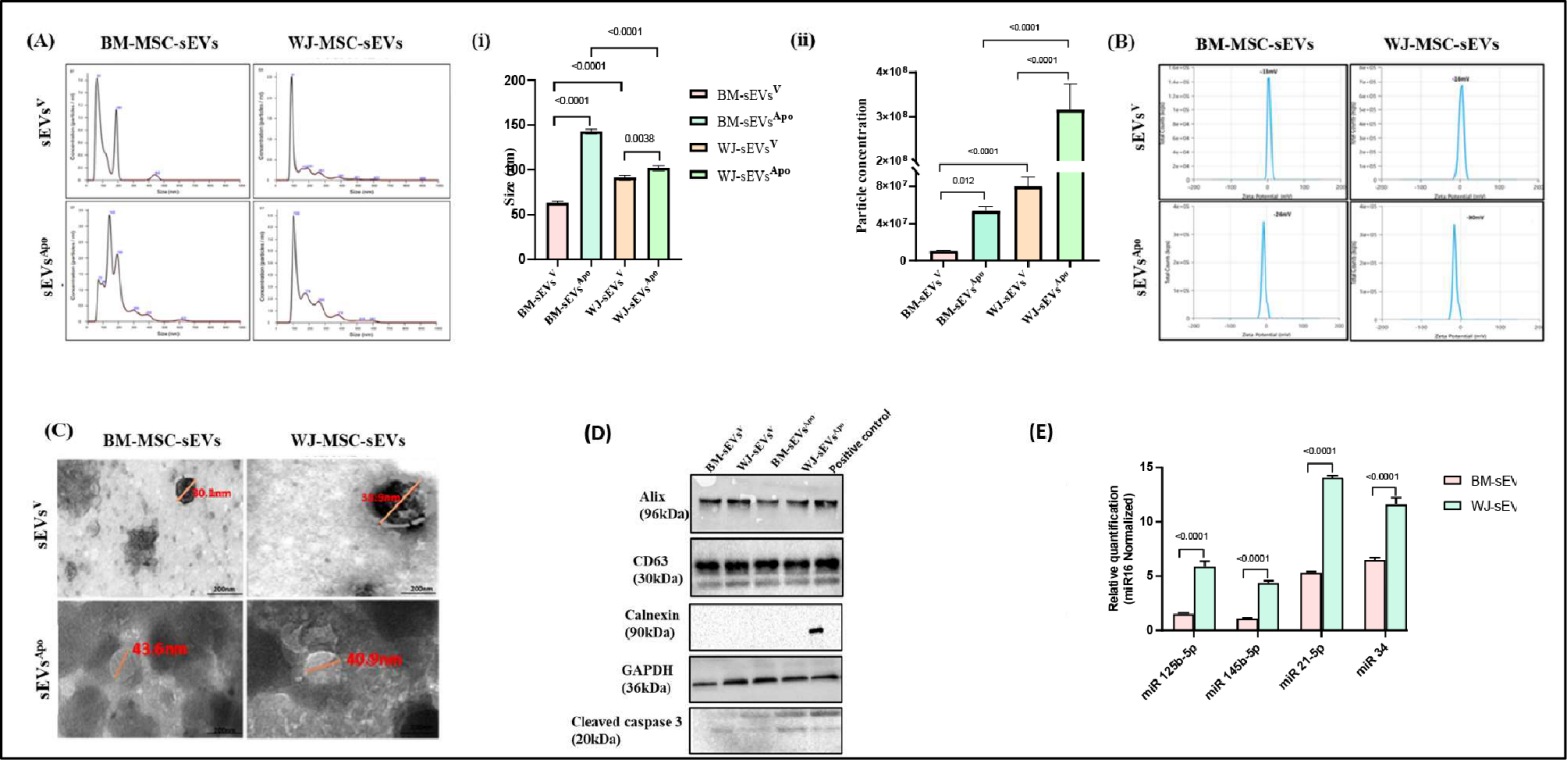
Characterization of tissue-specific (BM/WJ) human MSC-derived sEVs^V^ and sEVs^Apo^ for (**A**) Size distribution (i) & Particle concentration (ii) analysis using Nanoparticle Tracking Analysis (NTA) (**B**) Surface charge distribution using Zeta Sizer (**C**) Morphological analysis by Transmission Electron microscopy (TEM) (Scale Bar = 200 nm) (**D**) Western blot shows the presence of Alix, CD63, GAPDH, Cleaved Caspase 3 & absence of calnexin. (**E**) Upregulated expression of hepatoprotective miRNA in sEVs^Apo^. Quantitative analysis of miR 125b-5p, miR 145b-5p, miR 21-5p, miR 34a in tissue-specific (BM/WJ) human MSC-derived sEVs^V^ and sEVs^Apo^ by qPCR Abbreviation- V: Viable; Apo: Apoptotic. Data are shown as mean ± SD; Statistical analysis: ****p < 0.0001. All experiments were done in triplicates. Abbreviation- V: Viable; Apo: Apoptotic

sEVs obtained from the conditioned medium of both tissue-specific MSCs^V^ and MSCs^Apo^ displayed analogous characteristics in accordance with the MISEV 2018 guidelines [28]. These features encompassed a cup-shaped morphology, indicating preserved membrane integrity as observed through TEM; a size below 200 nm, as identified by NTA; and the existence of surface tetraspanin CD63, cytoplasmic protein ALIX, along with the absence of Calnexin (sEVs negative marker), confirmed via western blotting (Fig. 2A-D). Additionally, cleaved caspase 3 was also detected in both tissue specific sEVs^Apo^, but not in sEVs^V^, confirming their respective apoptotic & viable origins [19] (Fig. 2D).

Dissecting into the results obtained from NTA analysis we found that BM-sEVs^Apo^ & WJ-sEVs^Apo^ showed a higher particle concentration (3.5 × 10^7^ and 2.5 × 10^8^ respectively) as compared to the BM-sEVs^V^ & WJ-sEVs^V^ (1.6 × 10^7^ and 9 × 10^7^respectively) (Fig. 2A i, ii); and demonstrated a significantly greater (p: ≤0.0001, p: ≤0.01) mean particle size (143 nm and 102 nm, respectively) as compared to the BM-sEVs^V^ & WJ-sEVs^V^ (63 nm and 91 nm, respectively).

Also, the membrane potential of BM-sEVs^Apo^ & WJ-sEVs^Apo^ was − 13mV and − 16 mV respectively, as compared to the membrane potential of their viable counterparts − 26mV and − 30mV (Fig. 2B) as observed via the ZetaPotential Analysis.

### sEVs^Apo^ are highly instrumental in driving macrophage polarization towards anti-inflammatory phenotype via efferocytosis

**Fig. 3:**
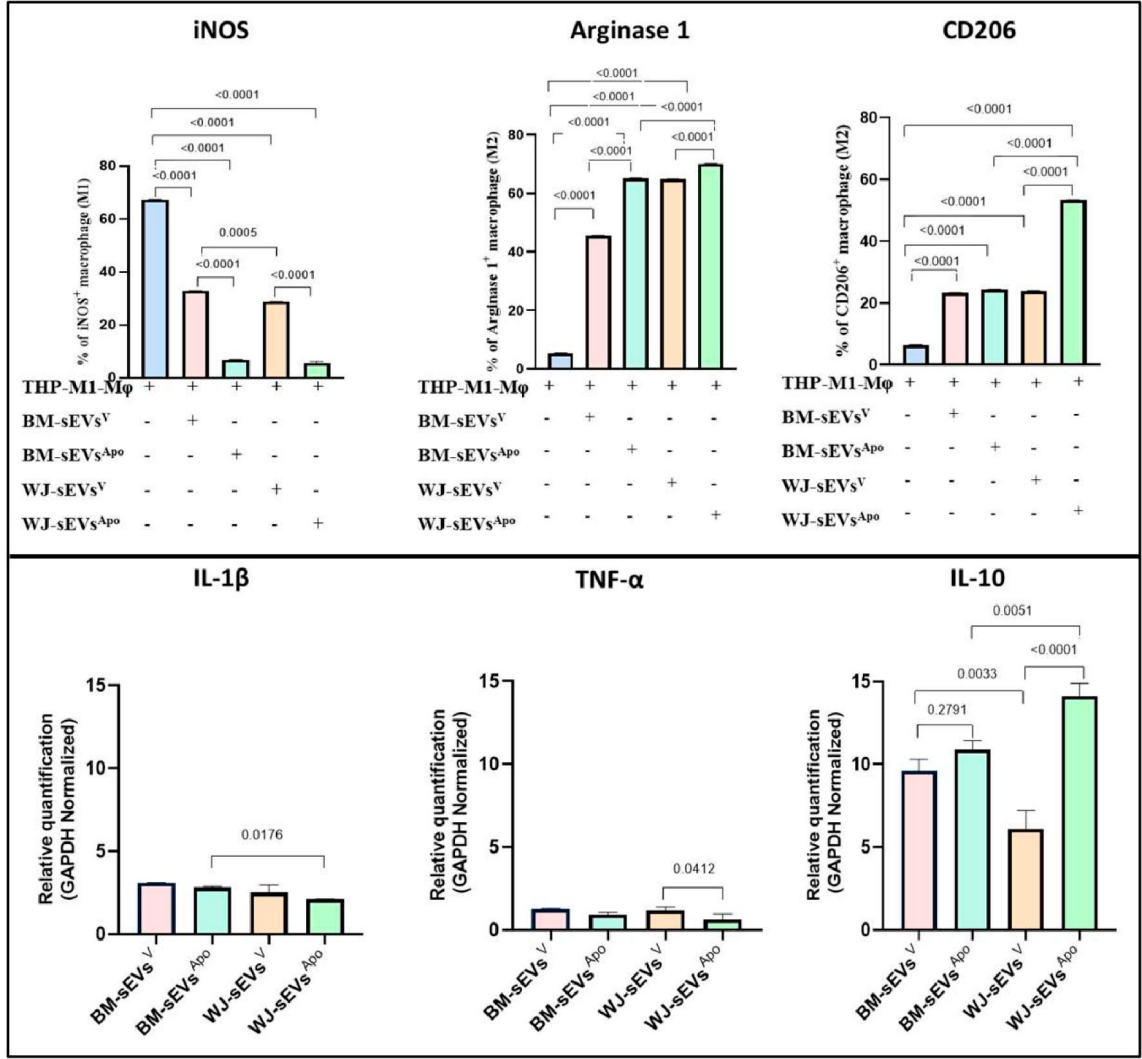
sEVs^Apo^ enhanced the polarization of M1 macrophages to M2 macrophages. (**A**) Bar graph showing the expression of (i) iNOS (M1), (ii) Arginase, and (iii) CD206 (M2) after the treatment of tissue-specific (BM/WJ) human MSC-derived sEVs^V^ and sEVs^Apo^. (**B**) Relative mRNA expression of (i) IL-Iβ, (ii) TNF-α, and (iii) IL-10 after the treatment of tissue-specific (BM/WJ) human MSC-derived sEVs^V^ and sEVs^Apo^ by qPCR. Data shown as mean ± SD; Statistical analysis: ****p < 0.0001. All experiments were done in triplicates. Abbreviation- V: Viable; Apo: Apoptotic

To validate their immunomodulatory functionality, LPS-activated THP1-induced macrophages were co-cultured with tissue-specific sEVs^V^ & sEVs^Apo.^ Herein, we observed a significant polarization of M1 macrophages towards the M2 phenotype after sEVs^Apo^ treatment compared to the sEVs^V^ treatment in both tissue sources. The polarization was assessed in terms of a significant increase in Arginase & CD206 expression (M2 markers; p: ≤0.0001), and the concurrent decrease in iNOS (M1 marker; p: ≤0.0001) (Fig. 3, S3).

In terms of the tissue source, it was observed that sEVs derived from WJ-MSCs^Apo^ were more efficient in macrophage polarization as compared to the BM-MSCs^Apo^. This was ascertained by the significant increase in anti-inflammatory M2 markers Arginase (59.68%; p:≤0.0001 and 64.51%; p:≤0.0001 in BM-sEVs^Apo^& WJ-sEVs^Apo^ respectively) & CD206 expression (24.43%; p:≤0.0001 and 53.20%; p:≤0.0001 in BM-sEVs^Apo^& WJ-sEVs^Apo^ respectively) (Fig. 3, S3).

A similar trend was also observed in the gene expression level of pro-inflammatory (IL-1β & TNFα) & anti-inflammatory (IL-10) cytokines, wherein the latter was significantly decreased, and the later was significantly increased in sEVs^Apo^ as compared to sEVs^V^ (p: ≤0.001) (Fig. 3).

**Fig. 4:**
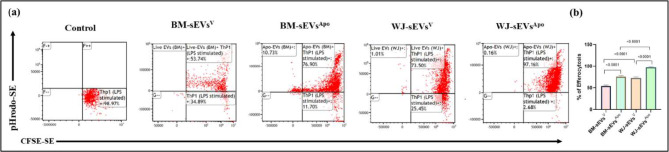
Efferocytosis of sEVs^Apo^ enhanced by macrophages. A representative (**a**) dot plot and (**b**) bar graph showing the magnitude of efferocytosis of tissue-specific (BM/WJ) human MSC-derived sEVs^V^ and sEVs^Apo^ by macrophage. Data shown as mean ± SD; Statistical analysis: ****p < 0.0001. All experiments were done in triplicates. Abbreviation- V: Viable; Apo: Apoptotic

To understand the aforementioned heightened functional capabilities of sEVs^Apo^, particularly those derived from WJ-MSCs^Apo^, we assessed the efferocytotic activity of both tissue-specific sEVs^V^ and sEVs^Apo^. The results revealed that BM-sEVs^Apo^& WJ-sEVs^Apo^ exhibited significantly increased efferocytosis activity (73.13% and 94.93%; p: ≤0.001 respectively) compared to BM-sEVs^V^ & WJ-sEVs^V^ (29.06% and 36.68%, respectively), thereby explaining the higher immunomodulation via WJ-sEVs^Apo^ (Fig. 4).

### sEVs^Apo^ suppress T-Cell proliferation and enhance Treg induction as one of their Immunomodulatory mechanisms

For the evaluation of the immunomodulatory potential of sEVs^Apo^, we investigated their role on the proliferation of immune-inductive T cells. Additionally, we assessed whether sEVs^Apo^ could induce a more effective polarization of T cells towards regulatory T cells (Tregs) compared to sEVs^V^ [18].

**Fig. 5:**
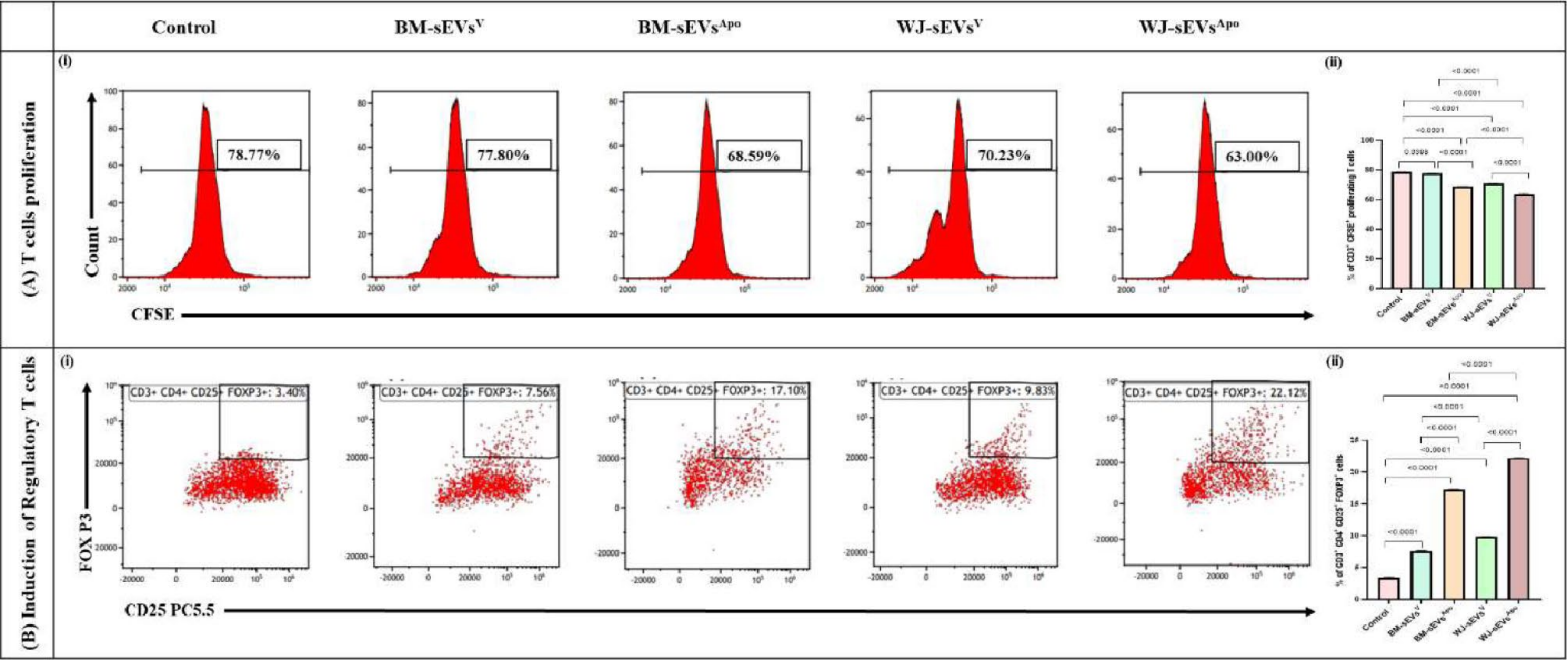
Apoptotic sEVs mediated suppression of T cell proliferation and induction of regulatory T cells. (**A**) Immunosuppressive effect of tissue-specific (BM/WJ) human MSC-derived sEVs^V^ and sEVs^Apo^ on the proliferation of T cells using CFSE-based T cell proliferation assay. (**B**) Induction of FoxP3 + Regulatory T cells after treatment with tissue-specific (BM/WJ) human MSC-derived sEVs^V^ and sEVs^Apo^. Data are shown as mean ± SD; Statistical analysis: ****p < 0.0001. All experiments were done in triplicates. Abbreviation- V: Viable; Apo: Apoptotic

It was found that sEVs^Apo^ significantly reduced CD3^+^ T cell proliferation & enhanced their induction into Tregs as compared to sEVs^V^ in both the tissue sources (p: ≤0.0001). Furthermore, WJ-sEVs^Apo^ were able to confer a greater reduction in T-cell proliferation as compared to the BM-sEVs^Apo^ (63% & 68.59%; p: ≤0.0001 respectively) (Fig. 5A). Moreover, Treg induction was significantly enhanced in WJ-sEVs^Apo^ (22.12%) as compared to BM-sEVs^Apo^ (17.10%) (Fig. 5B).

### Efficient mitochondrial ROS alleviation by sEVs^Apo^ as compared to their viable counterparts

**Fig. 6:**
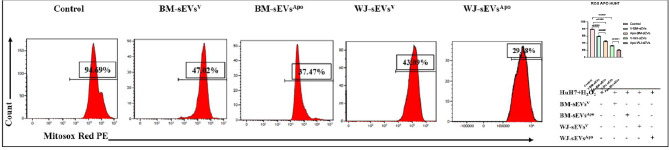
Regulation of Intracellular ROS level in HuH7 cells after sEVs^Apo^ treatment. Quantitation of intracellular ROS levels in HuH7 cells after the treatment of tissue-specific (BM/WJ) human MSC-derived sEVs^V^ and sEVs^Apo^ by qPCR. Data shown as mean ± SD; Statistical analysis: ****p < 0.0001. All experiments were done in triplicates. Abbreviation- V: Viable; Apo: Apoptotic

For the evaluation of the oxidative stress reduction potential of sEVs, we treated the hydrogen peroxide-induced HuH7 cells with the tissue-specific sEVs^V^ & sEVs^Apo^ for 24 h, post which the oxidative stress was evaluated using Mitosox staining via flow cytometry. Our results indicated that oxidative stress was significantly reduced in BM-sEVs^Apo^ (38%, p: ≤0.01) and WJ-sEVs^Apo^ (34%, p: ≤0.001) treated groups as compared with the BM-sEVs^V^ (45%, p: ≤0.05%) and WJ-sEVs^V^ (42%, p: ≤0.001) treated groups (Fig. 6). This demonstrated that apoptotic induction to the MSCs can significantly enhance the reparative capabilities of sEVs derived from them, and inhibit oxidative stress-induced injury [29, 30].

**Fig. 7:**
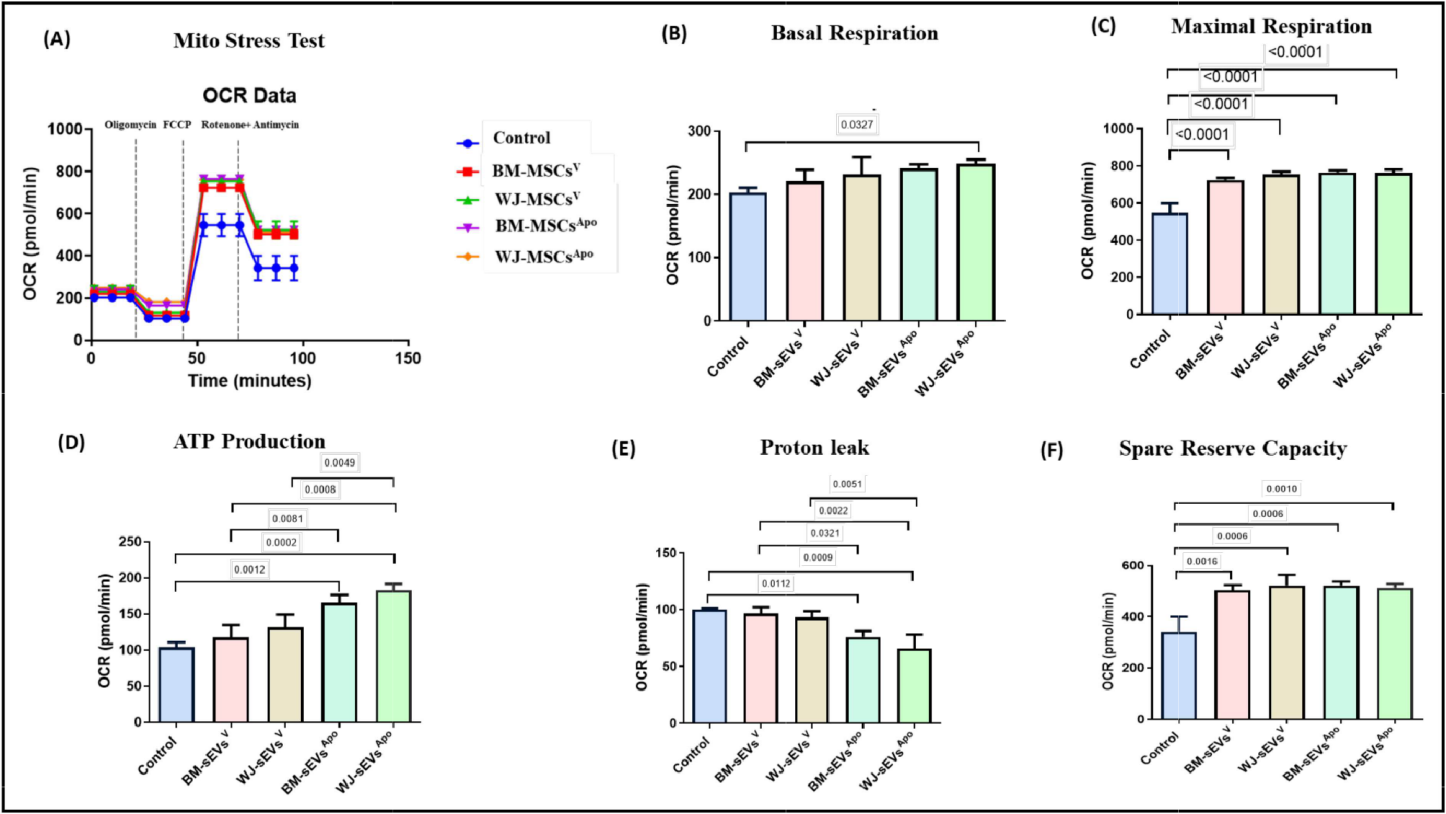
Mitochondrial parameters related to bioenergetics of tissue-specific MSC-sEVs^Apo^. (**A**) Real-time changes in oxygen consumption rate (OCR) with subsequent treatment with oligomycin, FCCP and rotenone and antimycin A. sEVs^Apo^ improves the mitochondrial respiratory parameters including (**B**) Basal respiration, (**C**) Maximal respiration (**D**) ATP production, (**E**) Proton Leak, (**F**) Spare Reserve capacity of HuH7 cells. Data shown as mean ± SD; Statistical analysis: ****p < 0.001. All experiments were done in triplicates. Abbreviation- V: Viable; Apo: Apoptotic

In order to further specifically analyze the effect of both sEVs^V^ & sEVs^Apo^ on mitochondrial health, we performed the seahorse extracellular flux mitochondrial stress assay. Upon treatment of HuH7 cells with sEVs from all groups (tissue-specific; viable and apoptotic MSCs), it was observed that the basal respiration rate, the maximal respiration rate & the spare respiratory reserve were improved in contrast to the untreated group. However, concerning the mitochondrial functionality, as assessed by the ATP production capacity and the proton leak, it was observed that sEVs^Apo^ were significantly improving the ATP production & reducing the proton leak in the cells as compared to the sEVs^V^, regardless of the tissue source. It was also found that WJ-sEVs^Apo^ were observed to significantly enhance basal respiration as compared to other groups (p: ≤0.05) (Fig. 7), as compared to BM-sEVs^Apo^.

### Immunomodulatory MiRNAs are enriched in sEVs^Apo^

To identify the mechanism for the enhanced immunomodulatory & antioxidant functionality of sEVs^Apo^, we sought to evaluate the content of sEVs^Apo^ in comparison to sEVs^V^, in terms of miRNAs known to target antioxidant & immunomodulatory signaling pathways.

Therefore, we assessed the expression of miR125b-5p, miR145a-5p, and miR34 in both the tissue-specific sEVs^V^ and sEVs^Apo^ via qPCR. The results showed a significant increase in the packaging of all 4 regenerative miRNAs in sEVs^Apo^ in comparison with sEVs^V^ in both BM & WJ (p: ≤0.001). Specifically, there was a 3.5-, 3.2-, 9-, and 5-fold higher upregulation of miRNA 125b-5p, miR 145b-5p, and miR 34 respectively in WJ-sEVs^Apo^ as compared to BM-sEVs^Apo^ (Fig. 1E).

### MSCs‑sEVs^Apo^ alleviates CCl4‑induced mice liver fibrosis

**Fig. 8:**
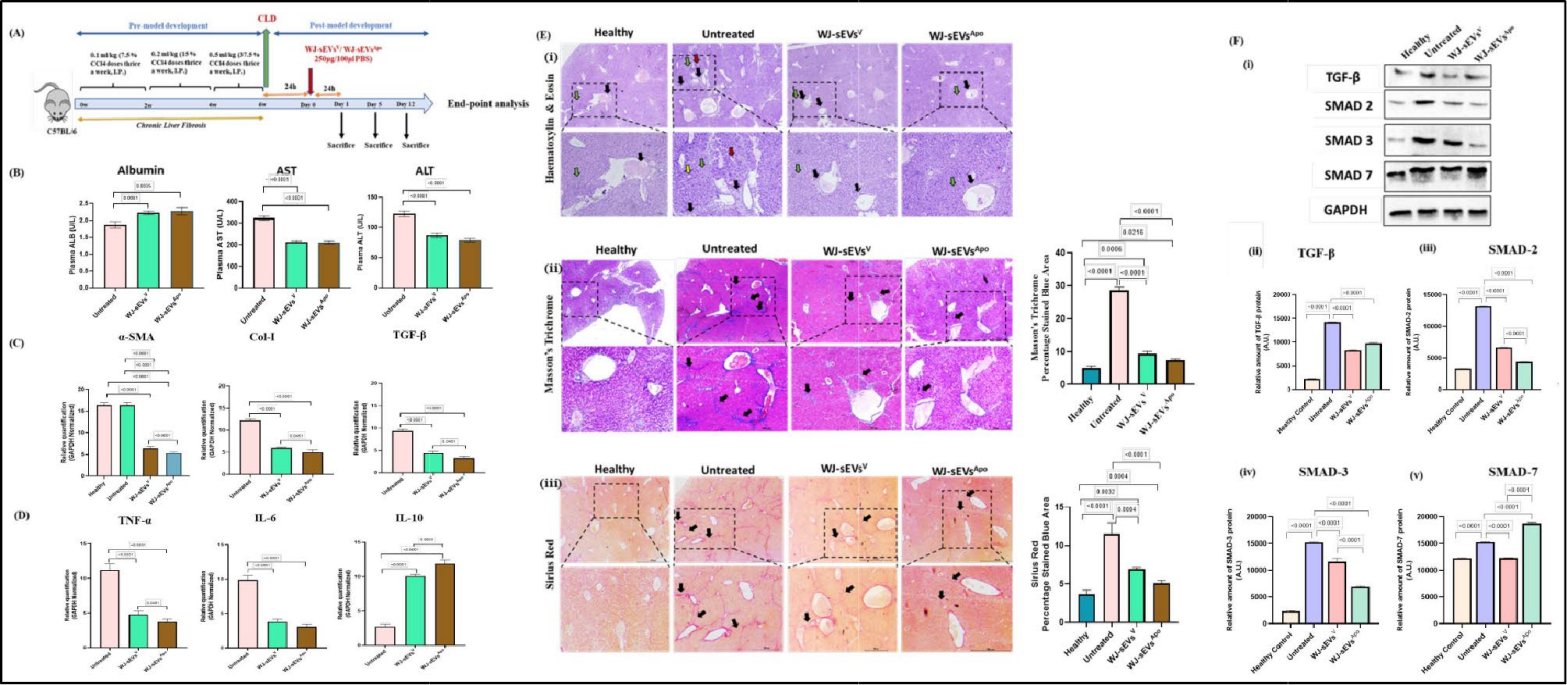
Intravenous infusion of sEVs^Apo^ ameliorates CCl4-induced liver fibrosis at Day 12 (**A**) Timeline representing WJ-sEVs^V^ and WJ-sEVs^Apo^ treatment in mice and sacrifice for downstream analysis (**B**) The plasma levels of Albumin, ALT and AST after the treatment of WJ-sEVs^V^ and WJ-sEVs^Apo^. (**C**) Quantitative analysis of collagen content positive area in SR staining. Relative qPCR analysis for fibrotic genes (Col-I, TGF-β, α-SMA), (**D**) pro-inflammatory gene (TNF-α, IL-6), and anti-inflammatory gene (IL-10) expression after the treatment of WJ-sEVs^V^ and WJ-sEVs^Apo^ (**E**) Histopathological analysis of mice liver sections by (i) H&E staining (black arrows: inflammation); (ii) Masson’s Trichrome staining (black arrows: collagen deposition); (a) Quantitative analysis of collagen content positive area in MT staining. (iii) Sirius Red Staining (black arrows: Collagen fibers); (a) Quantitative analysis of collagen content positive area in SR staining. Histological grading and fibrosis assessment using the Ishak scoring system (0–6) (**F**) WJ-sEVs^Apo^ regulates the TGF-β/Smad2/Smad3/Smad7 signaling pathway. (i) The protein expression levels of TGF-β, p-Smad2, p-Smad3, and Smad7 in the liver tissues after the treatment of WJ-sEVs^V^ and WJ-sEVs^Apo^ were determined by western blot analysis. The relative protein expression of (ii) TGF-β, (iii) p-Smad2, (iv) p-Smad3, and (v) Smad7. Data shown as mean ± SD; Statistical analysis: ****p < 0.0001. All experiments were done in triplicates (*N* = 3). Abbreviation- V: Viable; Apo: Apoptotic

Carbon tetrachloride (CCl4) is extensively employed as a hepatotoxic agent in liver fibrosis models due to its ability to replicate the pathophysiological features of human fibrosis. In this study, a CCl4-induced murine model was utilized to evaluate the therapeutic effects of WJ-sEVs^V^ and WJ-sEVs^Apo^ on fibrosis (Fig. 8A). Blood biochemistry analyses revealed a significant reduction in alanine aminotransferase (ALT) and aspartate aminotransferase (AST) levels, accompanied by an increase in plasma albumin levels following WJ-sEVs^Apo^ treatment (Fig. 8B). Histopathological examination of liver sections demonstrated a marked reduction in portal and lobular inflammation post WJ-sEVs^Apo^ treatment, along with decreased collagen bridging and deposition, as evidenced by Masson’s trichrome (MT) staining, compared to untreated controls. Quantitative analysis of MT-stained areas indicated that WJ-sEVs^Apo^ have greater potential for reversing fibrosis. Consistent findings were obtained with Sirius Red staining, where the percentage of the red-stained regions was significantly reduced after WJ-sEVs^Apo^ treatment relative to WJ-sEVs^V^ (Fig. 8E). Additionally, histological grading and fibrosis assessment using the Ishak scoring system (0–6), conducted by a liver pathologist blinded to the treatment groups, revealed that WJ-sEVs^Apo^ administration led to a substantial reduction in hepatocellular necrosis, fatty infiltration, vascular congestion, inflammation, sinusoidal dilatation, and fibrosis (Figure S4). The Ishak score for the WJ-sEVs^Apo^ -treated groups decreased from F4 to F1, indicating regression of collagen deposition in portal areas and a reduction in fibrous septa when compared to untreated groups (Table S1).

### Apoptotic-small extracellular vesicles (sEVs^Apo^) modulate fibrotic and inflammatory gene expression, leading to the transcriptional alterations that mitigate liver fibrosis

The gross changes in mice liver tissue after sEVs^Apo^ treatment were further analyzed for transcriptional changes by real-time PCR. Collagen I, α-SMA, TGF-β, TNF-α, IL-6, and IL-10 expression was evaluated on day 12. We have also evaluated the expression of IL-10, an anti-inflammatory cytokine, and IL-6, TNF-α, a pro-inflammatory cytokine. IL-10 expression was upregulated after sEVs^Apo^ treatment while TNF-α and IL-6 were downregulated (Fig. 8D). WJ-sEVs^Apo^ were observed to significantly target the expression of immuno-modulatory genes like TNF-α, IL-6, and IL-10, and reduce the expression of key fibrotic genes like Collagen I, α-SMA, and TGF-β (Fig. 8C). However, both sEV groups were able to ameliorate the fibrotic conditions significantly.

### WJ-sEVsApo exhibits superior anti-fibrotic effects in the CCl4-Induced liver fibrosis model by modulating TGF-β/Smad2/Smad3 pathway

Following confirmation of the antifibrotic effects of sEVs^Apo^, we explored the mechanisms underlying fibrosis reversal. WJ-sEVs^Apo^ demonstrated the ability to inhibit the upregulation of TGF-β1 protein in fibrotic livers and suppress the expression of downstream p-Smad2 and p-Smad3 genes (Fig. 8F). In the CCl4-induced liver fibrosis model, phosphorylation and nuclear translocation of Smad2/3 were significantly increased. However, WJ-sEVs^Apo^ treatment effectively inhibited these processes.

Western blot analysis further revealed that WJ-sEVs^Apo^ treatment suppressed the upregulation of TGF-β1 and p-Smad2/3 expression while increasing Smad7 expression in fibrotic livers (Fig. 8F). These results suggest that WJ-sEVs^Apo^ effectively suppresses the TGF-β/Smad signaling pathway, preventing excessive ECM accumulation and reversing fibrosis progression.

## Discussion

MSCs are currently leading the way in regenerative medicine therapeutics [31]. One of the major mechanisms of action for MSCs to carry out their functions is via the release of extracellular vesicles [32]. Several reports also suggest that MSCs undergo apoptosis post-infusion to exhibit their reparative functions [11]. Thereby, certain studies have investigated the role of MSCs derived apoptotic vesicles in regeneration [22]. In lieu of that, our study aimed to comprehensively assess the regenerative and immunomodulatory potential of tissue-specific MSC-derived sEVs, derived under apoptotic (MSC-sEVs^Apo^) and naïve (sEVs^V^) conditions, both in vitro and in vivo, via a liver injury model.

Notably, existing literature has often encompassed the Apo-EVs population, including both small and large EVs, as well as apoptotic bodies, however to the best of our knowledge, our study is the first to focus specifically on the Apo-sEVs population (size < 200 nm) [22].

While Apo-sEVs were found to maintain the basic sEV characteristics, interestingly NTA analysis demonstrated an increase in the average particle size and total concentration of MSCs^Apo^-sEV derived both tissue sources, considering an identical number of MSCs (1 × 10^5^) and volume of conditioned media (3 ml), to start with. We speculate that the increase in size could be due to the enrichment in cargo thereby also conferring a heightened functionality to sEVs^Apo^ over sEVs^V^, with BM-sEVs being better than WJ-sEVs, as observed by subsequent in vitro assays. This is further in line with the previous finding by Liang et al. (2017), who confirmed the increase in size of HEK 293T-sEVs upon miR26a enrichment [33]. As per our current understanding, this is the first study reporting the increase in size of MSCs-sEVs^Apo^, possibly due to cargo enrichment.

Furthermore, the increase in particle number is consistent with the previous research indicating that apoptotic cells release a greater number of microparticles compared to viable cells [18]. The speculated mechanism for the increased secretion has been suggested to involve the activation of scramblases during apoptosis, triggering membrane budding [26]. Caspase-3 has been found to cleave Rho-associated protein kinase (ROCK I), which leads to the remodelling of actin and microtubules present at the plasma membrane, therefore resulting in membrane blebbing and EV formation. However, the precise mechanism for the heightened secretion needs further elucidation [27, 34].

Apoptosis induction may alter the membrane composition of sEVs, including changes in surface markers, lipids, and membrane-associated proteins that facilitate enhanced cellular uptake and receptor-mediated signaling. Studies have shown that apoptotic cells and their derivatives (EVs) exhibit modified phospholipid profiles, such as increased phosphatidylserine exposure (22), which could enhance recognition and internalization by recipient immune cells, thus augmenting their immunomodulatory effects.

Additionally, apoptosis-induced changes in sEV surface proteins (such as PD-L1and cleaved caspase-3) could contribute to their superior efficacy [20, 22, 35]. We have also considered that the increased concentration and altered biophysical properties of Apo-MSC-sEVs may improve their biodistribution, cellular targeting, and therapeutic efficacy. These modifications have been highlighted in track changes mode in the revised manuscript.

We further investigated whether sEVs^Apo^ imitate the immunomodulatory potential of their parent cells [18, 36, 37, 38, 39–40]. Several reports have suggested that MSCs-sEVs exert immunomodulation by undergoing engulfment via efferocytosis into macrophages, thereby instructing their polarization by virtue of the miRNA cargo they carry [18, 36–38] [41].

Our study demonstrated that sEVs^Apo^ were more efficient in polarization of macrophages, as well as efferocytotic activity, as compared to their viable counterparts. This observation is supported by a study from Patil et al., (2021) in myocardial ischemic injury, which suggested that MSCs-EVs were able to enhance the efferocytosis of apoptotic cardiomyocytes, thereby leading to an increased macrophage polarization activity towards the anti-inflammatory phenotype [18, 42].

In comparison between the tissue sources, WJ-sEVs was faring significantly better than BM-sEVs. This is further in line with a study from Zhu Y et al., suggesting the better macrophage polarization capabilities of WJ-MSCs [32].

Another immunomodulatory mechanism exhibited by MSCs-sEVs is the inhibition of T-cell proliferation (CD3^+^) and promoting their differentiation into regulatory T cells (CD3^+^ CD4^+^ CD25^+^ FoxP3^+^) [39, 40]. We observed that sEVs^Apo^ were found to promote this polarization significantly better as compared to naïve EV. This is supported by a previous finding by Chen et al., 2019, who observed that the administration of Apo-EVs derived from primary murine thymocytes & Jurkat cells were able to promote regulatory T cell induction & suppress the production of T-helper cells [18]. Furthermore, WJ as a source was found to be more potent in exhibiting this activity.

Both of the above compelling findings pertaining to the potential of sEVs^Apo^ towards macrophage polarization & T-reg induction, affirm the superior immunomodulatory potential of sEVs^Apo^ when compared to their viable counterparts. This robust outcome not only underscores the pivotal role of sEVs^Apo^ in immunomodulation over sEVs^V^, but also emphasizes the heightened tissue-specific functionality of WJ-MSCs over BM-MSCs as a source.

Heightened inflammatory milieu also leads to the induction of reactive oxygen species (ROS), which impairs the mitochondrial activity and results in oxidative stress via disruption of the electron transport chain [26, 43, 44]. A large body of evidence has shown the capabilities of MSCs-sEVs^V^ in ROS alleviation, however there is still a dearth of knowledge about the same with sEVs^Apo^ [22, 29, 45, 46] [47, 48].Our study demonstrated that sEVs^Apo^ exhibited notable efficacy in reducing mitochondrial ROS, potentially restoring the cellular homeostasis [49]. [In terms of the tissue source, WJ- exhibited a heightened ability to reduce oxidative stress, in comparison with BM. To further elucidate the specific mechanism of alleviation of ROS, we evaluated the mitochondrial bioenergetics of sEV treated groups [49–53]. While, all sEVs treated groups were instrumental in improving mitochondrial fitness, sEVs^Apo^ were more efficient in reducing the proton leak & enhancing the ATP production, suggesting that sEVs^Apo^ are more potent in enhancing mitochondrial energetics, functionality, and reducing mitochondrial damage.

Moreover, WJ-sEVs^Apo^ demonstrated a significant enhancement in the basal respiration rate, highlighting their superior capacity for ROS reduction compared to BM-MSCs. This effect may be attributed to their ability to support recipient cells in meeting the energy demands required for reparative processes [29, 48].

Conclusively, we established the superior immunomodulatory and antioxidative capabilities of sEVs^Apo^ over sEVs^V^ and WJ- over BM-sEVs.

We further examined the miRNA content of sEVs^Apo^ in comparison to sEVs^V^ in a tissue-specific context. This analysis aimed to uncover potential mechanisms underlying their enhanced functionality and to validate our earlier observations of increased sEVs^Apo^ size, which we hypothesized to be associated with miRNA cargo enrichment. Previous studies have demonstrated the incorporation of miRNA 125b-5p, miRNA 145b-5p, miRNA 21-5p, and miRNA 34 in tissue-specific sEVs^V^. These miRNAs are known to regulate key cellular signalling pathways, including NF-κB, JAK/STAT, MAPK, and NOTCH, which are primarily involved in immunomodulation and ROS mitigation [2, 26, 54–56]. In our study, we observed that the enrichment of all these miRNAs was significantly upregulated in WJ-sEVs^Apo^, thereby evidencing their heightened functional relevance, as well as the increase in size [49–52]. 

Notably, this study demonstrates the potential of apoptosis induction as a strategy to enhance the immunomodulatory & regenerative capabilities of MSCs-derived sEVs. It also presents an approach towards developing an economical and minimally manipulated regenerative modality. Although the current findings were validated in an in vitro liver injury model, its impact is far and wide, showing hope for other diseases as well.

Further studies are required to elucidate the changes in the expression of fibrotic markers following WJ-sEVs^Apo^ treatment to assess its comparative regenerative efficacy. Our study demonstrated that WJ-sEVs^Apo^ treatment effectively reduced liver damage markers, such as ALT (alanine aminotransferase) and AST (aspartate aminotransferase), which are typically elevated in liver injury. Additionally, plasma albumin levels increased, suggesting an improvement in liver synthetic function after WJ-sEVs^Apo^ treatment. Histological analysis indicated that WJ-sEVs^Apo^ significantly improved several liver pathology parameters, including hepatocellular necrosis, fibrosis, inflammation, and hepatocyte ballooning. Notably, sinusoidal dilatation, a marker of liver vascular injury, was completely reversed with WJ-sEVs^Apo^ treatment. Masson’s trichrome (MT) staining and Sirius Red staining were employed to assess collagen deposition and fibrosis severity. Both techniques revealed that WJ-sEVs^Apo^ treatment led to a significant reduction in fibrotic tissue compared to the untreated group. The Ishak scoring system, commonly used to evaluate liver fibrosis, also showed improvement, with WJ-sEVs^Apo^ treatment reducing the fibrosis score from F4 to F1, indicating a reversal of fibrosis and regression of collagen in the liver portal areas. Notably, as compared to the untreated group, there was a remarkable reduction in the expression of fibrotic markers including α-SMA, TGF- β, and Collagen I in both the sEVs groups. Interestingly, WJ-sEVs^Apo^ were observed to have a superior capacity to ameliorate liver fibrosis as indicated by significant downregulation of Collagen I as compared to that of WJ-sEVs^V^ treatment groups. This is an important finding as Collagen I is usually highly upregulated in human liver cirrhotic conditions amongst all other collagen types and thus identified as dangerous collagen. These results in line with a report by Cui Y et al. where Embryonic Stem Cell-derived MSC-sEVs were instrumental in improving the hepatocyte viability and reducing both apoptosis and the expression of pro-fibrotic molecules, such as collagen, α-SMA, and TIMP-1, while increasing the expression of collagenases, such as matrix metalloproteinase MMP-9 and − 13 [34]. Our findings also pointed towards the anti-inflammatory effect of WJ-sEVs^Apo^ that was evidenced by changes in the expression of inflammatory genes when evaluated in mice tissue. Moreover, our in-vitro preliminary studies have also supported our observations that LPS-induced inflammatory response was altered after WJ-sEVs^Apo^ exposure.

To further investigate the precise signaling pathway behind the improved regenerative abilities of WJ-sEVs^Apo^ in the liver fibrosis model, we evaluated the transcription factors of the TGF-β/SMAD signaling pathway, a key driver of fibrogenesis. Notably, in the untreated group, the protein expression levels of pro-fibrotic mediators TGF-β, p-Smad2, and p-Smad3 were markedly elevated compared with the healthy group in the CCL4‑induced liver fibrosis model, consistent with the hyperactivation of this pathway during fibrosis progression. Treatment with WJ-sEVs^V^ and WJ-sEVs^Apo^ significantly reduced the expression of TGF-β, p-Smad2, and p-Smad3 compared with the untreated group, highlighting its ability to suppress the pro-fibrotic cascade. Furthermore, WJ-sEVs^Apo^ uniquely enhanced Smad7 expression, promoting negative feedback within the signaling pathway and further attenuating fibrogenesis. While WJ-sEVs^Apo^ exhibited the suppressive effects on TGF-β, p-Smad2, and p-Smad3 levels primarily, WJ-sEVs^V^ also exhibited a suppressive effect to a notable extent.

The findings of this study align with prior research on the antifibrotic potential of mesenchymal stem cell-derived small extracellular vesicles in liver fibrosis [26]. Placental-derived MSC-sEVs have been shown to downregulate TGF-β1 and TGF-β2 expression, thereby reducing hepatic stellate cell activation and extracellular matrix deposition in fibrotic livers (Zhao et al., 2019). Similarly, adipose-derived MSC-sEVs have demonstrated a capacity to attenuate fibrosis by inhibiting TGF-β signalling (Li et al., 2020). This study advances these findings by demonstrating that WJ-sEVs^Apo^, a bioengineered variant of Wharton’s Jelly-derived small extracellular vesicles (sEVs), offers enhanced efficacy in modulating the TGF-β/Smad signalling pathway. This effect underscores the superior performance of WJ-sEVs^Apo^, potentially attributed to its optimized molecular cargo, which is more potent than those in unmodified MSC-sEVs [35]. These findings suggest that WJ-sEVs^Apo^ may provide superior anti-fibrotic activity, potentially due to enhanced bioactivity and targeted modulation of signalling pathways.

While existing studies primarily focus on assessing the regenerative and immunomodulatory abilities of sEVs^V^, to the best of our knowledge, our study stands out as the first to compare their regenerative potential with that of sEVs^Apo^, across 2 different MSC tissue sources, Bone Marrow & Wharton’s Jelly at one platform. Notably, WJ-sEVs^Apo^ faired significantly better in all the domains of in vitro & in vivo functionality evaluated in the current study. Furthermore, our study is the first to focus on a homogeneous population of Apo-sEVs (size < 200 nm), eliminating the vesicles possibly having detrimental components as a by-product of apoptosis.

## Conclusion

Our findings highlight a manifold enhancement in the therapeutic and regenerative potential of sEVs^Apo^, positioning them as a promising cell-free therapy, especially in conditions where MSCs penetration is challenging, such as brain and heart disorders. The increased yield of sEVs upon apoptotic induction further emphasizes its potential for translation, addressing the significant roadblock of low sEVs yield in clinical applications. However, further investigations into the other specific mechanisms of immunomodulation, and contents of sEVs^Apo^ in various disease scenarios are crucial to unlocking their clinical potential. Nonetheless, this study acts as a stepping stone for further investigation in this domain, underscoring the necessity for exploration and research to delve into the underlying mechanisms behind the regenerative and immunomodulatory potential of sEVs^Apo^.

## Electronic supplementary material

Below is the link to the electronic supplementary material.

Supplementary Material 1

Supplementary Material 2

## Data Availability

The data that support the findings of this study are available from the corresponding author upon reasonable request.

## Abbreviations

MSC: Mesenchymal Stem Cells
sEVs: Small Extracellular Vesicles
CCl4: Carbon tetrachloride
TGF-β: Transforming growth factor-β
IL-6: Interleukin-6
IL-10: Interleukin-10
TNF-α: Tumor Necrosis Factor-α
AST: Aspartate Aminotransferase
ALT: Alanine Aminotransferase
ALB: Albumin
Apo: Apoptotic
V: Viable
