# Supplementary material for: Uncovering the bequeathing potential of apoptotic mesenchymal stem cells via small extracellular vesicles for its enhanced immunomodulatory and regenerative ability
Source: Stem Cell Res Ther. 2025 Jun 7;16:290. doi: 10.1186/s13287-025-04370-x (PMC12145648; doi:10.1186/s13287-025-04370-x)
Supplement: Supplementary file 1 — Supplementary Material 1 [file 13287_2025_4370_MOESM1_ESM.docx]

**Supplementary figure 1**


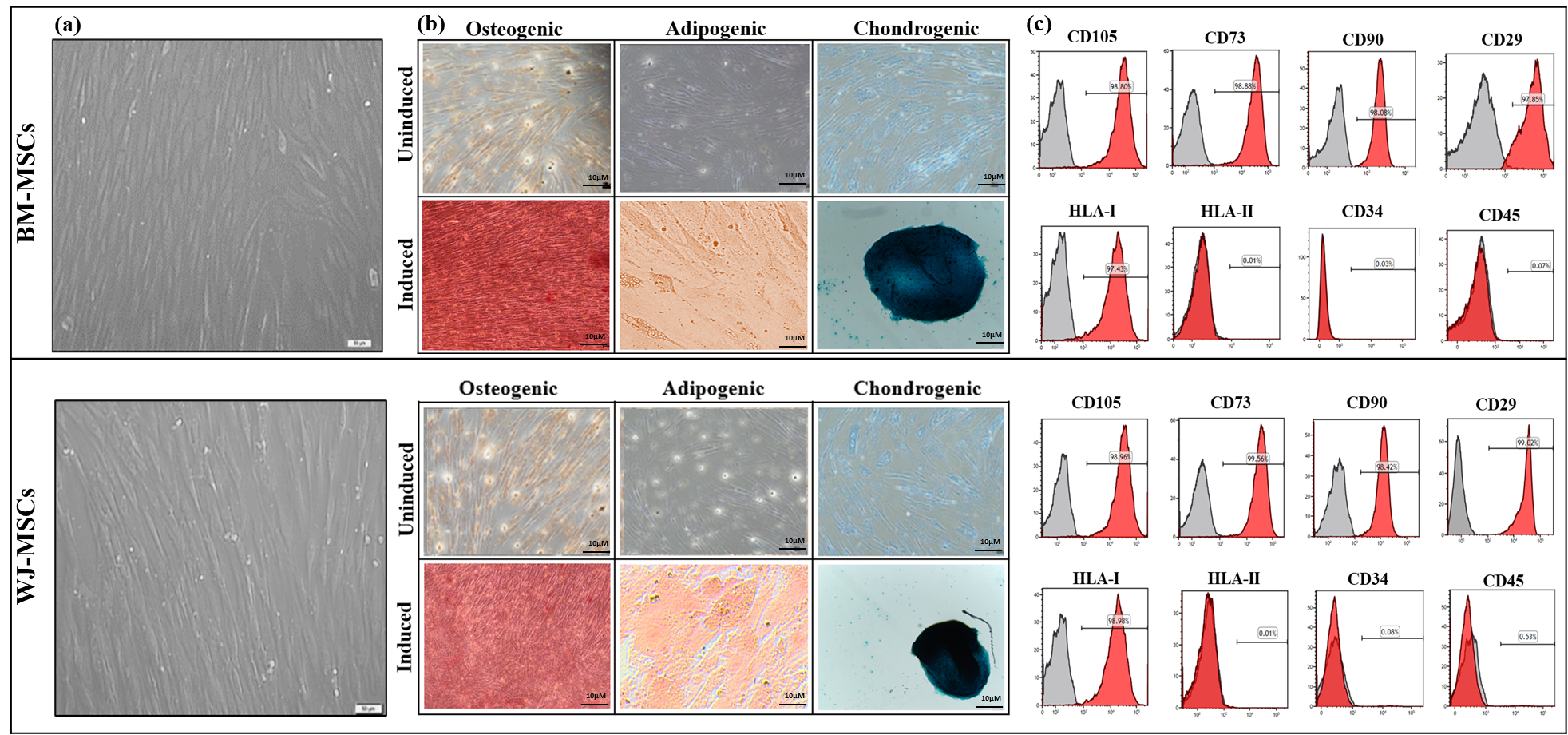


**Figure S1:** Characterization of tissue-specific (BM/WJ) human MSCs as per ISCT guidelines (a) Representative images of fibroblast-like morphology of BM-MSCs and WJ- MSCs (Scale bar: 100µm). (b) Trilineage differentiation of BM-MSCs and WJ-MSCs into Osteocytes (21 days of induction), Adipocytes (28 days of induction), and Chondrocytes (14 days of induction) (Scale bar: 100µm). (c) Surface marker profiling of BM-MSCs and WJ- MSCs via flow cytometry.

**Supplementary figure 2**


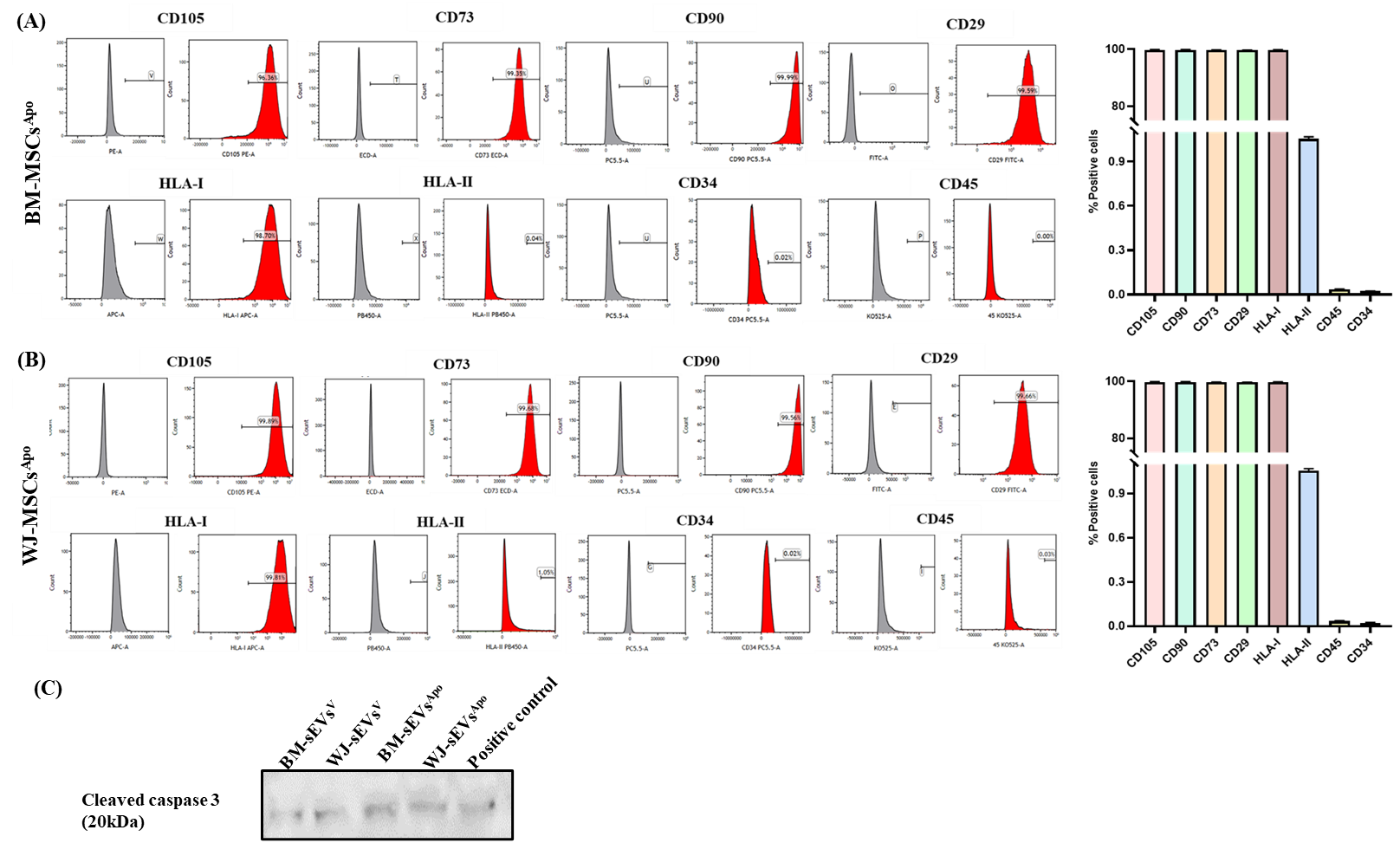


**Figure S2:** Characterization of tissue-specific Apo (BM/WJ) human MSCs as per ISCT guidelines. Flow cytometric analysis of (A) BM-MSCs^Apo^ and (B) WJ-MSCs^Apo^ for positive surface markers CD90, CD 105, CD29, CD73, and HLA-I and negative surface markers HLA-II and CD34/45. (C) Western blot shows the presence of Cleaved Caspase 3. Abbreviation- Apo: Apoptotic

**Supplementary figure 3**


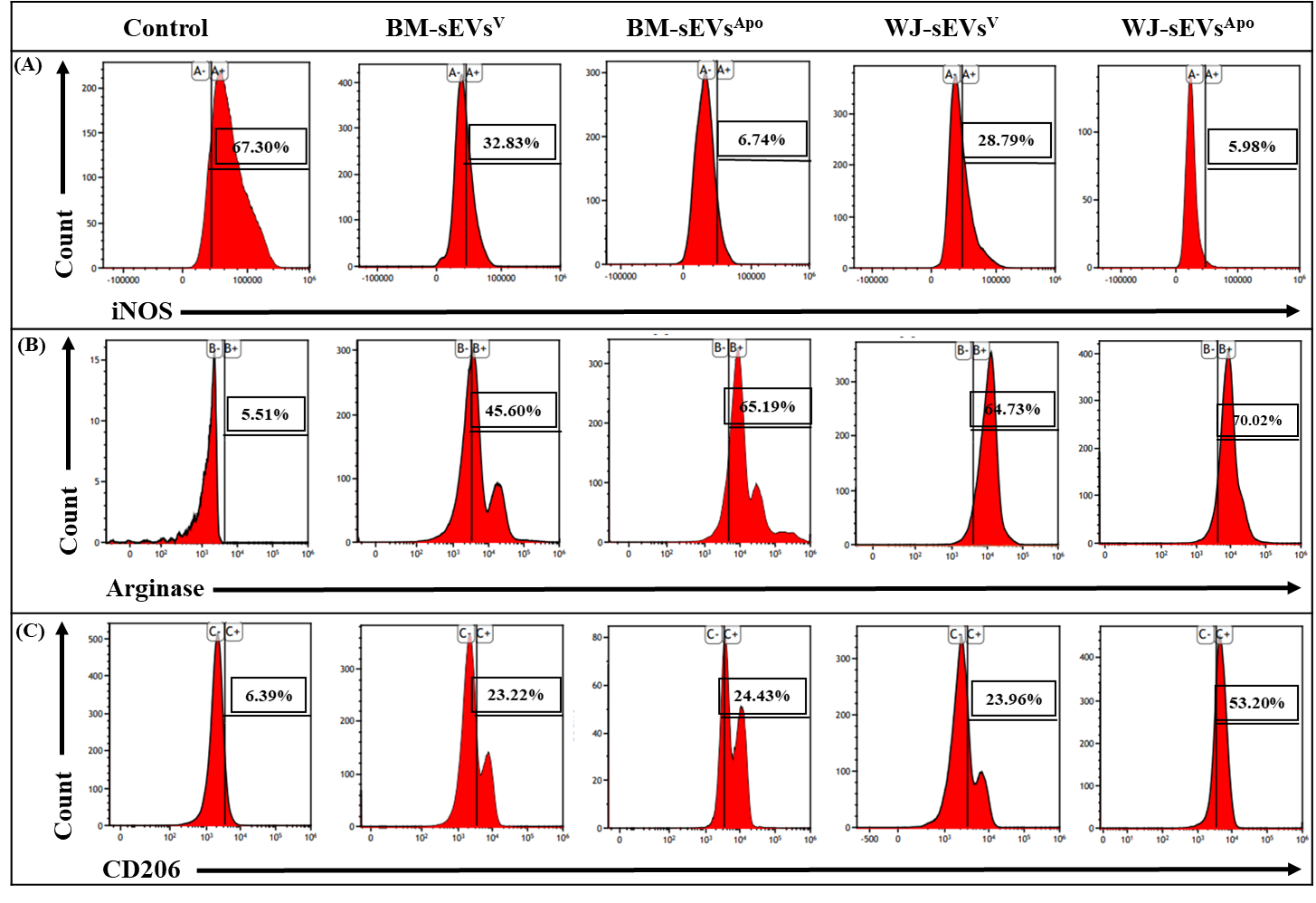


**Figure S3.** sEVs^Apo^ enhanced the polarization of M1 macrophages to M2 macrophages. (A) Flow cytometry analysis shows the expression of (A) iNOS (M1), (B) CD206, and (C) Arginase-1 (M2) after the treatment of tissue-specific (BM/WJ) human MSC-derived sEVs^V^ and sEVs^Apo^.

**Supplementary figure 4**

**
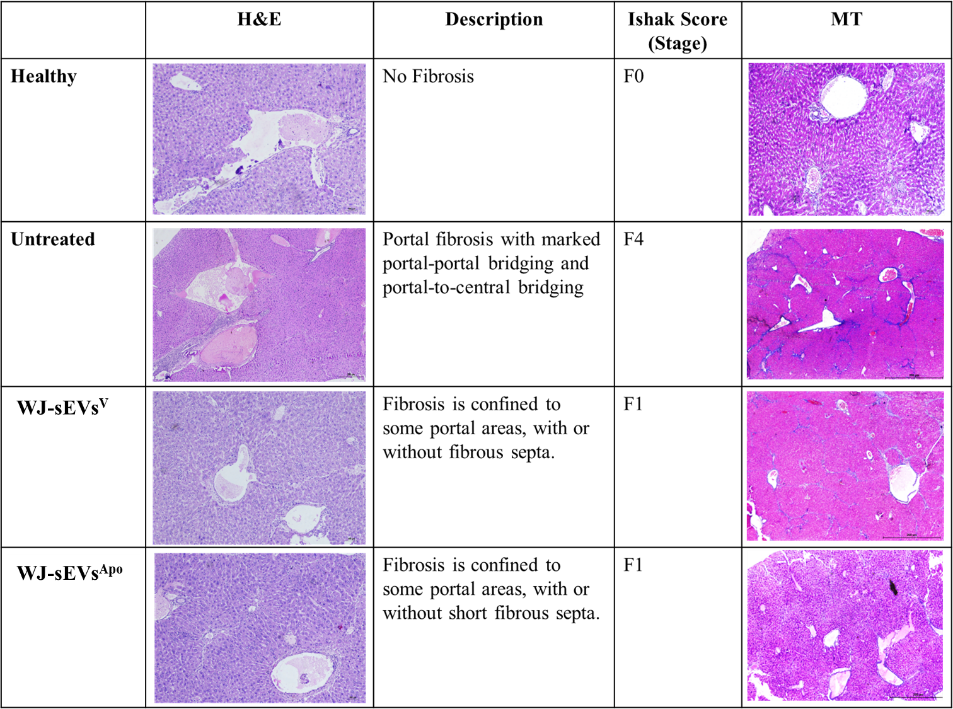
**

**Figure S4.** Histological scoring of liver fibrosis regression based on the ISHAK or Metavir scoring systems.

**Supplementary Figure 5**

**
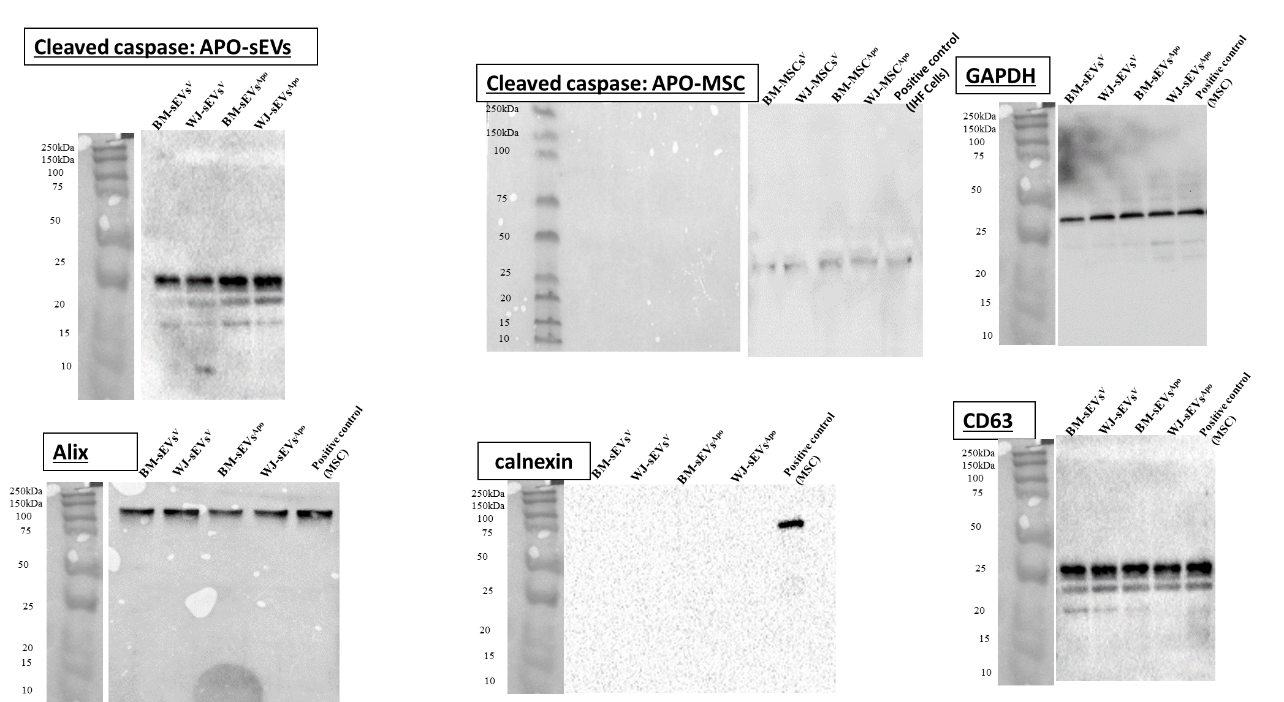
**

**Figure S5:** Full-length blots represent the expression of Cleaved caspase-3, GAPDH, ALIX, CALNEXIN, CD63 protein in tissue-specific (BM/WJ) human MSC-derived sEVs^V^ and sEVs^Apo^. And Cleaved caspase-3 in MSC^Apo^ (BM/WJ)

**Supplementary Figure 6**

**
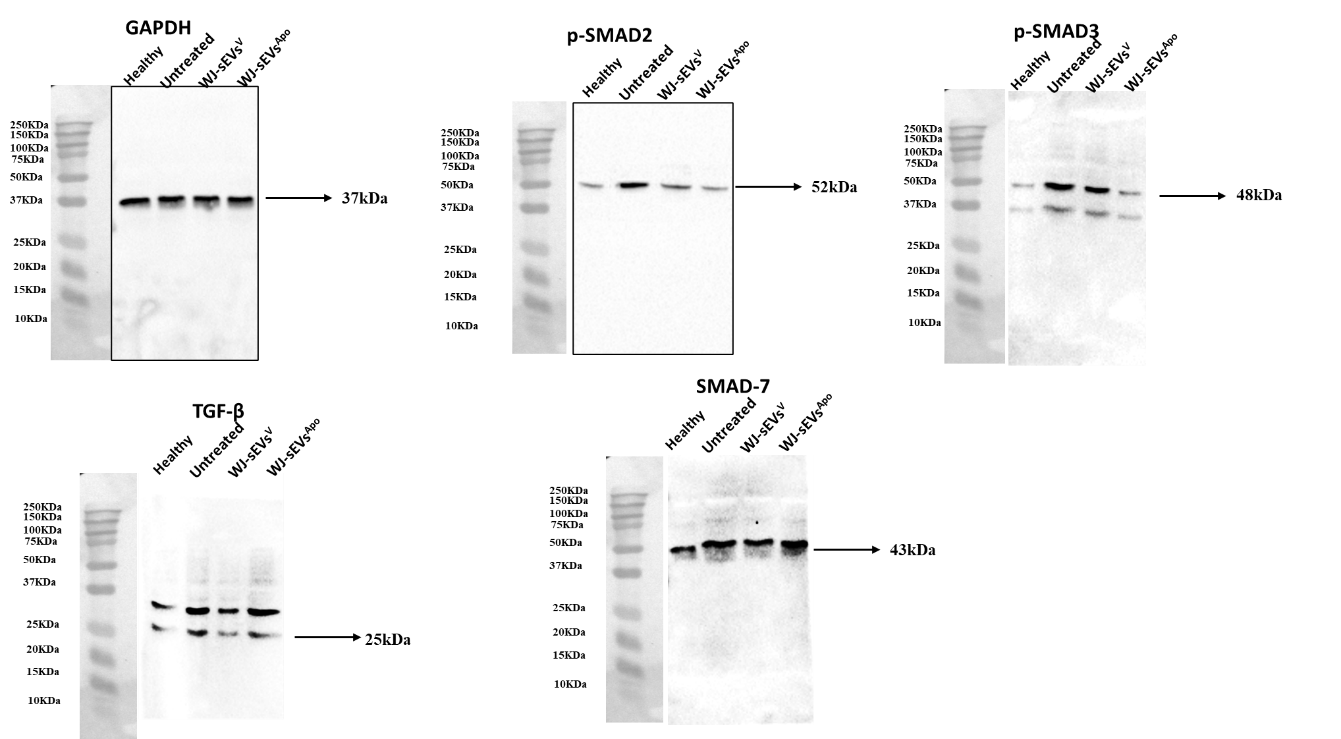
**

**Figure S6:** Full-length blots represent the expression of GAPDH, p-SMAD2, p-SMAD 3, TGF-β, SMAD7 protein in healthy liver tissue, CLD liver (Untreated), WJ-sEVs^V^ treated , and WJ-sEVs^Apo^ treated liver tissue.

**Graphical abstract**

**
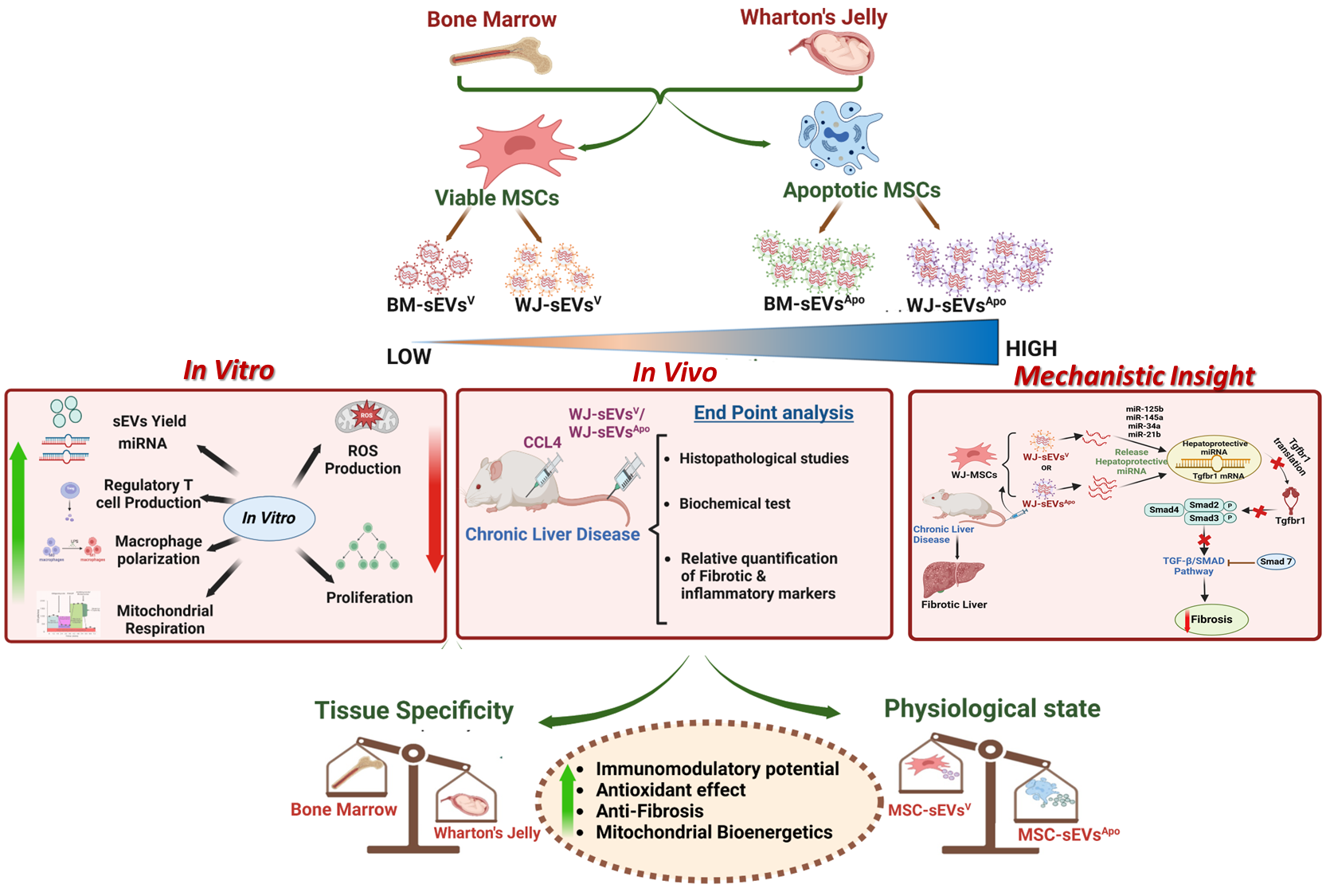
**
