# Supplementary material for: Uncovering the bequeathing potential of apoptotic mesenchymal stem cells via small extracellular vesicles for its enhanced immunomodulatory and regenerative ability
Source: Stem Cell Res Ther. 2025 Jun 7;16:290. doi: 10.1186/s13287-025-04370-x (PMC12145648; doi:10.1186/s13287-025-04370-x)
Supplement: Supplementary file 2 — Supplementary Material 2 [file 13287_2025_4370_MOESM2_ESM.docx]

**Table S1: Histological analysis of liver section**

| **S.No.** | **Assessment parameters** | **Untreated** | **WJ-sEVs^V^** | **WJ-sEVs^Apo^** |
| --- | --- | --- | --- | --- |
| 1 | Hepatocellular necrosis | ++ | + | + |
| 2 | Fibrosis | +++ | ++ | + |
| 3 | Inflammation | +++ | + | + |
| 4 | Hepatocyte ballooning | +++ | ++ | + |
| 5 | Sinusoidal dilatation | ++ | + | + |

+: minimum; ++: mild; +++: maximum

**Table S2: List of Antibodies**

| **S.No.** | **Antibody** | **Company** | **Catalogue Number** |
| --- | --- | --- | --- |
|  | CD90 | Becton Dickinson, USA | 555597 |
|  | CD73 | Becton Dickinson, USA | 550257 |
|  | CD105 | eBiosciences, USA | 17-1057 |
|  | CD29 | eBiosciences, USA | 14-0299 |
|  | HLA-ABC | Becton Dickinson, USA | 555555 |
|  | HLA-DR | Becton Dickinson, USA | 555560 |
|  | CD34/45 | Becton Dickinson, USA | 341071 |
|  | CD63 | Abcam, USA | ab59479 |
|  | ALIX | Genetex, USA | GTX135282 |
|  | Calnexin | Genetex, USA | GTX13504 |
|  | Cleaved Caspase-3 | CST, MA | 9661T |
|  | GAPDH | Genetex, USA | GTX100118 |
|  | iNOS | eBiosciences, USA | 53-5920-82 |
|  | CD206 | eBiosciences, USA | 25-2069-42 |
|  | Arginase 1 | eBiosciences, USA | 17-3697-82 |
|  | CD3, CD4, CD8, CD25, and CD45 | Beckman Coulter, USA | 6607013 |
|  | TGFB | Affinity Biosciences, USA | AF0260 |
|  | SMAD4 | Affinity Biosciences, USA | AF5247 |

**Table S3: List of Primers**

| **S. No.** | **Gene** | **Primer sequence** | **Company** |
| --- | --- | --- | --- |
|  | IL-10 | 5’-TCTCCGAGATGCCTTCAGCAGA-3’  5’-TCAGACAAGGCTTGGCAACCCA-3’ | IDT |
|  | IL-6 | 5’-AGACAGCCACTCACCTCTTCAG-3’  5’-TTCTGCCAGTGCCTCTTTGCTG-3’ | IDT |
|  | TNF-α | 5’-CTCTTCTGCCTGCTGCACTTTG-3’  5’-ATGGGCTACAGGCTTGTCACTC-3’ | IDT |
|  | IL-1β | 5’-CCACAGACCTTCCAGGAGAATG-5’  5’-GTGCAGTTCAGTGATCGTACAGG-3’ | IDT |
|  | IL-1β | 5’-CCACAGACCTTCCAGGAGAATG-5’  5’-GTGCAGTTCAGTGATCGTACAGG-3’ | IDT |
|  | GAPDH | 5’-GACAAGCTTCCCGTTCTCAG-3’  5’-GACAAGCTTCCCGTTCTCAG-3’ | Sigma-Aldrich |
|  | Mouse GAPDH | 5’GICTTCACCACCATGGAGAAGG-3’  5′- CTAAGCAGTTGGTGGTGCAGGA- 3’ | IDT |
|  | Mouse Collagen-I | 5′ -TGACTGOAAGAGCOGAGAGTA-3’  5′- GACOGCTGAGTAGGGAACAC-3’ | IDT |
|  | Mouse α-SMA | 5cGTGACATCGACATCAGGAAAGA −3  5c GATCCACATCTGCTGGAAGG-3’ | IDT |
|  | Mouse TNF-α | 5′- ACCCTCACACTCACAAACCA-3’  5′- ATAGCAAATCGGCTGACGGT-3’ | IDT |
|  | Mouse IL-10 | 5cTAACTGCACCCACTTCCCAG-3’  5’-AGGCTTGGCAACCCAAGTAA-3’ | IDT |
